# The green lacewing venom system and the complex mechanisms underlying its evolution

**DOI:** 10.1093/molbev/msaf326

**Published:** 2025-12-11

**Authors:** Marius F Maurstad, Iris Bea L Ramiro, Jan Philip Oeyen, Andy Sombke, Sebastian Büsse, Pedro G Nachtigall, Kjetill S Jakobsen, Eivind A B Undheim

**Affiliations:** Centre for Ecological and Evolutionary Synthesis, Department of Biosciences, University of Oslo, Oslo, Norway; Centre for Ecological and Evolutionary Synthesis, Department of Biosciences, University of Oslo, Oslo, Norway; Centre for Ecological and Evolutionary Synthesis, Department of Biosciences, University of Oslo, Oslo, Norway; Division of Biotechnology and Plant Health & Viruses, Bacteria and Nematodes in Forestry, Agriculture and Horticulture, Norwegian Institute of Bioeconomy Research (NIBIO), Oslo, Norway; Centre for Anatomy and Cell Biology, Cell and Developmental Biology, Medical University of Vienna, Vienna, Austria; Cytology and Evolutionary Biology, Zoological Institute and Museum, University of Greifswald, Greifswald, Germany; Centre for Ecological and Evolutionary Synthesis, Department of Biosciences, University of Oslo, Oslo, Norway; Centre for Ecological and Evolutionary Synthesis, Department of Biosciences, University of Oslo, Oslo, Norway; Centre for Ecological and Evolutionary Synthesis, Department of Biosciences, University of Oslo, Oslo, Norway

**Keywords:** comparative transcriptomics, genomics, molecular evolution, Neuroptera, venom gland, alternative splicing

## Abstract

Venom has independently evolved across many lineages, yet relatively few have been studied in detail, particularly among insects. Of these, Neuroptera (lacewings, antlions, and relatives) remain largely unexplored, despite being widespread with agriculturally important groups such as green lacewings. While adults are nonvenomous, neuropteran larvae are ferocious predators that use pincer-like mouthparts to inject paralyzing and liquefying venom to subdue and consume their prey. Here, we provide a comprehensive investigation of the venom system in Neuroptera by integrating a high-quality genome, long-read transcriptomes spanning all life stages, microCT-reconstruction of venom glands, tissue-specific expression analyses, venom proteomics, and functional assays of the common green lacewing *Chrysoperla carnea*. We provide a re-description of the neuropteran venom system, demonstrate the venom's insecticidal and cytotoxic activity, and show that the venom comprises diverse toxin gene families and is richer and more similar to the venom of antlions than previously proposed. We show that this toxin arsenal is the result of a multitude of evolutionary events that include co-option, recruitment following gene duplication, diversification of toxin-paralogs by gene duplication, and functional innovation of new paralogs through both small structural and large architectural changes. In addition, we find that alternative splicing of toxin genes is an important contributor to the biochemical arsenal, which is a mechanism rarely documented among venomous animals. Our results demonstrate how multiple genomic and evolutionary mechanisms together contribute to the emergence and evolution of a complex molecular trait, and provide new insights into the evolution of venom in insects.

## Introduction

Venoms are typically complex mixtures primarily composed of secreted proteins and peptides known as toxins that have evolved at least 100 times independently to facilitate key antagonistic interactions such as predation, feeding, and/or defense (Schendel et al. 2019; Jenner et al. 2025). On each of these occasions, the emergence of venoms and toxins was accompanied by the evolution of specialized venom-associated glands, delivery structures, and behaviors that together form a venom system (Schendel et al. 2019). Venom systems, therefore, represent excellent systems for exploring mechanisms underlying the evolution of novelty across levels of biological complexity (Schendel et al. 2025). Toxins usually evolve through gene duplications, neofunctionalization, and diversifying selection (Casewell et al. 2013), and are often convergently recruited from similar protein families across different taxa (Fry et al. 2009). Molecular mechanisms such as co-option (Martinson et al. 2017; Almeida et al. 2021; Barua and Mikheyev 2021) and changes in domain architecture (Pineda et al. 2020; Koludarov et al. 2023) also frequently contribute to toxin evolution. Although less common, alternative splicing (Ogawa et al. 2019; Gopalan et al. 2022; Pan et al. 2023; Ye et al. 2023) and horizontal gene transfer can also contribute to the arsenal of venoms (Moran et al. 2012; Undheim and Jenner 2021; Walker et al. 2023). As such, research into venom evolution and toxins provides valuable insights into fundamental questions about molecular evolution and functional innovation.

However, most venom research has traditionally focused on a handful of medically significant taxa, such as front-fanged snakes, leaving many venomous lineages largely or even completely unstudied. Insects, the most species-rich class of eukaryotes, encompass many lineages that have yet to be thoroughly investigated (Walker et al. 2018b). Among insects, there are 10 orders known to contain venomous species (Schendel et al. 2019; Jenner et al. 2025), and while we are rapidly gaining knowledge about the venom systems of Hymenoptera (Touchard et al. 2014, 2024; Schmidt 2016; Robinson et al. 2018, 2023; Dashevsky et al. 2023), most other venomous insect orders remain either largely or completely unstudied (Walker et al. 2018b). Among these underexplored taxa, Neuroptera stands out as a particularly intriguing lineage. As an ancient insect order dating back to the early Permian (∼281 Mya) (Vasilikopoulos et al. 2020) and consisting of around 6,000 extant species, Neuroptera offers a unique opportunity to study how molecular mechanisms shape venom composition and function over macroevolutionary timescales.

In contrast to their nonvenomous adult stage, larval neuropterans occupy a multitude of ecological niches and share specialized feeding structures in which the mandibles and maxillae connect to form a feeding tube, with the maxilla housing a venom canal (Beutel et al. 2010; Zimmermann et al. 2019; Li et al. 2022; Fischer et al. 2024). Within the maxillae, Neuroptera has evolved specialized medial and lateral venom glands, along with a third gland—the cephalic gland—which extends posteriorly from the basal part of the stylets within the head capsule (Fischer et al. 2024). These adaptations enable them to release venom that has been proposed to subdue their prey through extra-oral digestion (EOD) (Fischer et al. 2024). Historically, studies on Neuroptera venoms have focused on toxins produced by endosymbiotic bacteria in antlions (Myrmeleontidae) (Matsuda et al. 1995; Yoshida et al. 1999, 2001; Nishiwaki et al. 2004, 2007). These bacterial toxins, likely originating in the gut, have been shown to have paralytic effects when injected into prey species such as German cockroaches and common cutworms. Recent findings suggest that the venom of the antlion *Euroleon nostras* actually contains very few bacterial proteins and instead consists of toxins typically associated with animal venoms, such as phospholipase A2 (PLA_2_) (Fischer et al. 2024). In contrast, the venom of the common green lacewing *Chrysoperla carnea*—a species widely used in biological control due to its larvae's appetite for aphids and other agricultural pests—was found to include bacterial proteins alongside insect-derived components, while showing a reduced presence of putative venom toxins compared to the antlion (Fischer et al. 2024). However, despite these recent advances in our knowledge of the neuropteran venom system, our understanding of the molecular mechanisms contributing to the venom composition and toxin family evolution remains poor.

Here, we present a comprehensive investigation of the venom system of the widely distributed and agriculturally important *Chrysoperla carnea*. To characterize the diversity of venom components at both the gene and isoform levels, we sequenced the genome of a male individual and the transcriptomes of its offspring using long-read-based technologies. To understand the expression profiles of venom-producing tissues, we used micro-computed tomography (µCT) to characterize the morphology of maxilla-bound glands and guide dissections to properly assess tissue-specific expression patterns. Combining these approaches with a noninvasive method for the extraction of venom, we characterized the *C. carnea* venom using a proteotranscriptomic approach and assessed its cytotoxic and paralytic activities through functional assays. In contrast to previous findings, our results show that *C. carnea* possesses a potent and biochemically rich venom that has evolved through multiple molecular mechanisms, including “classical” toxin gene family recruitment and expansion, co-option of physiological genes, neofunctionalization through domain loss, and alternative splicing. Taken together, our results show that the venom arsenals of lacewings are richer and more potent than previously postulated and provide new insight into the molecular and evolutionary mechanisms that shape the venoms of insects.

## Results and discussion

### Genome assembly and annotation

Although a genome for the green lacewing already exists (Crowley 2021), genomic regions containing toxin genes have been shown to exhibit substantial inter-population structural variation in other venomous animals (Nachtigall et al. 2025b). To provide a high-resolution description of the venom composition of *C. carnea*, we therefore sequenced and assembled the genome of one male *C. carnea* using PacBio long reads and used its offspring to generate a comprehensive Iso-Seq-based transcriptomic dataset covering all life stages, including egg, first-, second-, and third larval instars, as well as adult males and females. The genome assembly achieved a total contig length of ∼600 Mbp with a contig N50 of 37 Mbp and an L50 of 5 (SI Figure S1), implying that the first 5 contigs cover 50% of the genome even without any spatial information. The genome had a BUSCO completeness score of 97.4% using the Endopterygota odb10 dataset (SI Table S1). For comparison with the previously published reference genome by Crowley (2021), which comprises 1 X- and 5 autosomal-pseudochromosome scaffolds, see SI Table S2. By integrating the Iso-Seq dataset with RNA-sequencing of dissected tissues (see below) and the long-read genome assembly from the male individual, we produced a high-quality gene annotation for *C. carnea*. Analyzing the completeness of the annotated gene models achieved a 98% BUSCO completeness score using the Endopterygota odb10 dataset (SI Table S1).

### The green lacewing has an advanced venom system producing paralyzing and cytotoxic venom

**Figure 1:**
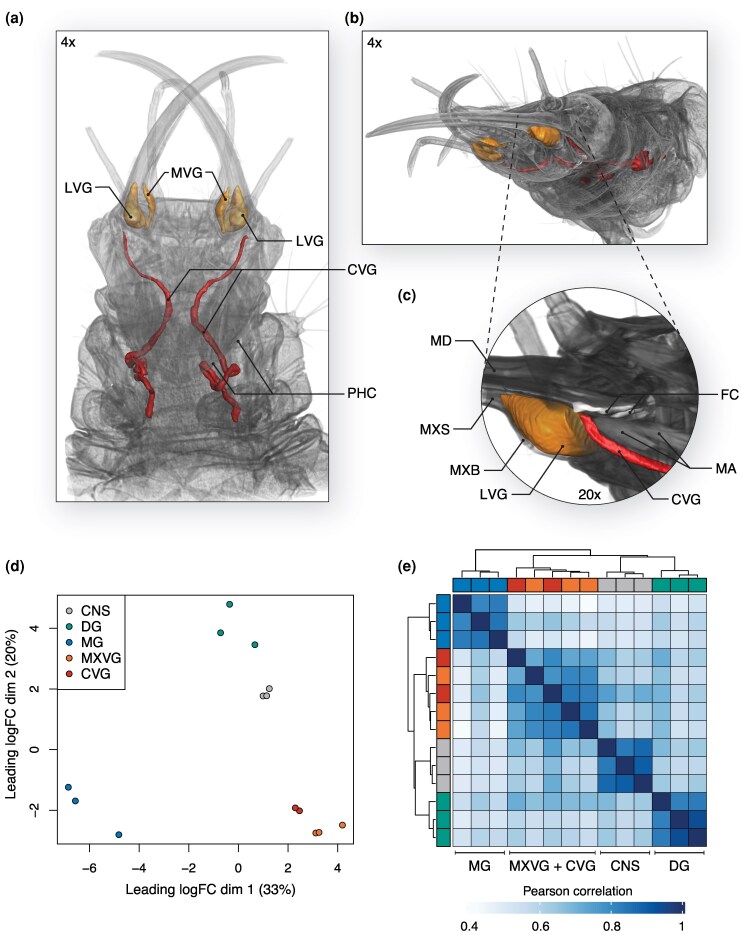
Morphological and transcriptomic characterization of the venom system in *C. carnea*. a) 3D reconstruction of the larval venom system from µCT scanning at 4× resolution viewed dorsally, showing the lateral (LVG) and medial venom glands (MVG) contained within the maxillary stylet (MXVG) and the cephalic venom gland (CVG) extending from the head through the neck and beyond the posterior head capsule (PHC) into the prothorax. b) Overview reconstruction shown from the ventral-lateral view at 4× resolution. c) Zoomed inset (20× resolution) of the lateral view of the venom system showing the mandible (MD), maxillary stylet (MXS), and maxillary stylet bulb (MXB) containing the LVG and MVG. The CVG passes below and lateral to the food canal space (FC, white) between the MD and MXS and connects to the MXB through a lumen (SI Figure S2). The mandibular abductor muscle (MA) is positioned above the CVG. d) Multidimensional scaling (MDS) and e) hierarchical clustering of transcriptomic data obtained from dissected tissues. The tissue samples are color-coded as in the MDS plot in panel d).

The cephalic gland in *C. carnea* has been described as a separate structure terminating in the head capsule and opening into the food canal (Fischer et al. 2024). However, our combination of micro-dissection and µCT shows that the cephalic gland connects through a venom duct to the maxillary stylet bulb (MXB), which contains the lateral venom gland (LVG) and the medial venom gland (MVG)—hereafter referred to as MXVG. This forms a closed venom system that is not connected to the food canal (Fig. 1a, SI Figure S2). The cephalic gland extends through the larval neck and into the thorax, representing a substantial elongation of the gland (Fig. 1a) compared to previously reported neuropteran families, where the cephalic gland terminates in the head capsule (Beutel et al. 2010; Zimmermann et al. 2019; Li et al. 2022). Based on these observations, we hereafter refer to the “cephalic gland” as the “cephalic venom gland” (CVG).

Given that the CVG connects to MXVG via a venom duct, we next examined whether these 2 venom-producing tissues exhibited similar expression profiles. To explore this, we performed RNA sequencing (RNA-Seq) in triplicate of 5 pooled individuals from µCT-guided tissue dissections, including the maxillary stylets containing the MXVG, the CVG, the central nervous system (CNS), midgut (MG), and the defense gland (DG; anal glands that produce a defensive secretion). We then leveraged our high-quality gene annotation data to perform gene-level differential expression analyses of the micro-dissected tissues. This approach revealed that the venom-producing tissues (MXVG and CVG) clustered together in both multidimensional scaling (MDS) (Fig. 1d) and hierarchical clustering analyses (Fig. 1e), indicating that they have similar expression profiles and likely have similar roles as venom-producing tissues. The elongation of the *C. carnea* CVG with respect to those of other neuropterans, and their expressional similarity to the MXVG, suggests *C. carnea* has a well-developed venom-producing system that plays important functions beyond the previously suggested primary role as extra-oral digestion of prey (Fischer et al. 2024).

**Figure 2:**
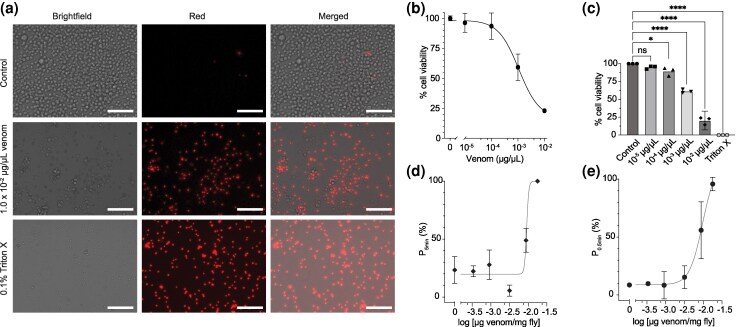
*C. carnea* has cytotoxic and paralytic venom. a) Images of *D. melanogaster* S2 cells stained with propidium iodide, which stains only lysed cells. 0.1% Triton X was used as a positive control. Scalebar represents 70 µm. b) A representative concentration response curve of venom tested on *D. melanogaster* S2 cells, with cell viability measured as percent viable cells relative to the negative control. Data points represent means ± SD c) Cell viability measured across multiple venoms from *C. carnea.* Experiments were conducted in technical triplicate, *n* = 3 biological replicates. Data points represent means ± CI95. **** indicates *P* < 0.0001 for comparing venom concentration *versus* control; * indicates *P* = 0.0104 for comparing 1 *×* 10*^−^*^4^ µg/µL venom *versus* control; ns: not significant (one-way ANOVA, Dunnett's test). d) A representative dose response curve of the venom causing paralysis in adult *D. melanogaster* (PD_50_ 9.4 ng/mg). *P*_5min_ indicates the percentage of flies that cannot climb at 5 min post-injection. Data points represent means ± SD, *n* = 3 trials. e) Paralytic activity of *C. carnea* venom measured as the percentage of adult *D. melanogaster* able to right themselves at 0.5 min after injection of venom (*P*_0.5min_). Data points represent means ± SD, *n* = 2 experiments (∼PD_50_ 9.6 ng/mg).

To test the alternative hypotheses that the venom of *C. carnea* is primarily involved in extraoral digestion or that it plays dual roles in both extraoral digestion and prey incapacitation, we performed functional assays of venom on *D. melanogaster* adult flies and *D. melanogaster* S2 cells. To avoid potential effects from inhibitors or unfinished post-translational activation that may be present in dissected venom or whole tissue homogenates, we used venom extracted by a nonharmful CO_2_-based method (SI Video S1). The venom showed cytotoxic activity against S2 cells, as indicated by a concentration-dependent reduction of cell viability (Fig. 2), with the highest concentration tested (10 ng/µL) reducing cell viability to 20.4% (Fig. 2a–c). Testing the venom in vivo in adult *D. melanogaster* resulted in a consistent paralytic phenotype (PD_50_ 9.4 ng venom/mg fly, pPD_50_ 2.027 ± 0.022), with flies recovering from injections of venom at lower doses (Fig. 2d and e). The paralytic activity of *C. carnea* venom is also similar to the toxicity phenotype that is observed during capture of live prey such as large *D. melanogaster* larvae (SI Video S2). Given the paralytic potency observed here and that the venom yields from individual larvae typically span 1 to 3 µg venom, we estimate that paralyzing an adult *D. melanogaster* requires roughly 1% to 20% of the total venom contained in the venom glands of an L3 *C. carnea* larva. Together, these results demonstrate that *C. carnea* possesses a well-developed venom system that is used to produce and deliver potent venom that not just digests but also paralyses prey.

### Venom proteomics and comparative tissue transcriptomics reveal the composition of the green lacewing venom

To comprehensively characterize the venom composition of *C. carnea*, we performed liquid chromatography–tandem mass spectrometry (LC–MS/MS) in triplicate on venom samples that were either reduced and alkylated or reduced, alkylated, and digested with trypsin. We searched the resulting mass spectra against our gene annotation and identified 1,134 proteins corresponding to 659 genes (SI Data S1). Many of these components lacked a signal peptide and/or were annotated as typical housekeeping proteins, such as proteins typically associated with the nucleus. Given the nondestructive method used for extracting venom, this pattern of protein composition suggests that venom is secreted via holocrine or apocrine mechanisms, or perhaps a combination of secretory mechanisms. For example, multiple secretory strategies have been described in centipedes, where both merocrine and apocrine-like secretion mechanisms are found within distinct secretory cell types (Schendel et al. 2025).

**Figure 3:**
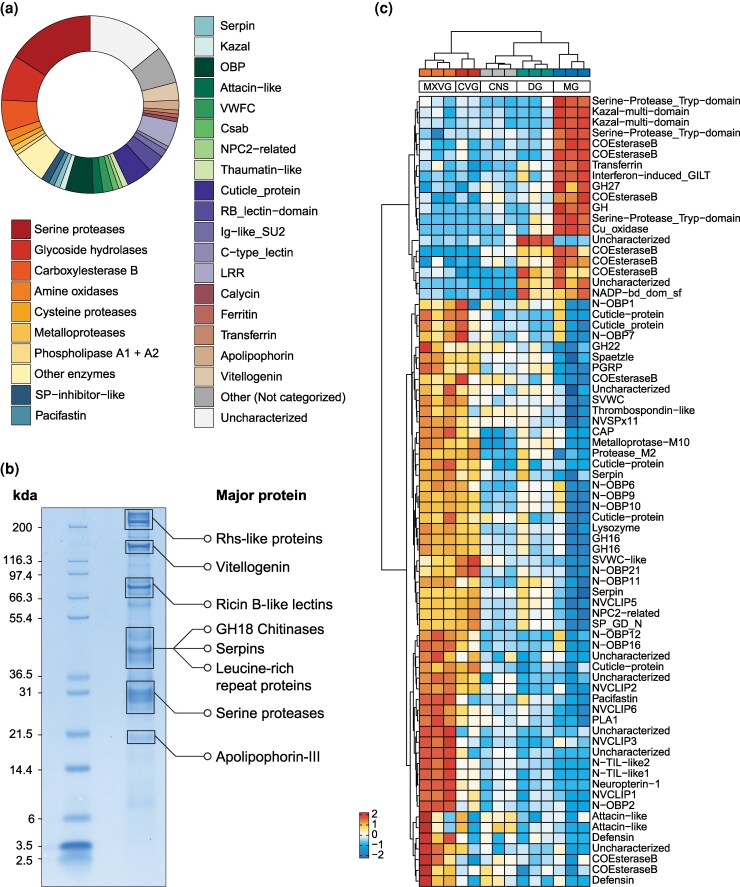
Venom composition, proteomic abundance, and tissue-specific expression of *C. carnea* venom components. a) Donut chart showing the composition of venom proteins categorized into major functional families based on the relative number of putative venom genes. b) SDS–PAGE gel of the *C. carnea* venom proteins, highlighting the major protein bands and their major protein identities based on LC–MS/MS analysis. c) Heatmap showing the differentially expressed genes among the 277 putative venom genes across the 5 tissues. Differential expression analysis was performed using the glmTreat function from the edgeR package, with a log_2_ fold-change threshold of log_2_(1.2) and a false discovery rate (FDR)-adjusted *P*-value < 0.05.

To retain only secreted candidate venom components, we filtered for genes where at least one isoform of a gene encoded a protein with a signal peptide, which is a characteristic of most venom proteins and peptides. The single exception to this filtering step was the inclusion of a Rhs-repeat containing protein that has previously been identified as a major component in mosquito venom (King et al. 2011; Liu et al. 2023). This approach returned 466 putative venom protein isoforms encoded by 281 genes, henceforth referred to as “venom genes” (Fig. 3a and b; SI Data S1). We also examined the expression of venom genes across our comparative transcriptomic dataset, filtering for genes upregulated in the venom-producing tissues compared to other tissues. Hierarchical clustering of the candidate venom gene expression revealed 2 major clusters: one set of genes upregulated in both the defense glands and the midgut and one set upregulated in the venom glands only (Fig. 3c, left dendrogram). The first major cluster subdivides into genes upregulated in both the defense glands and midgut (with the exception of one uncharacterized protein exclusive to the defense glands) and genes upregulated in the midgut. Of these subclusters, the latter probably represents minor venom components primarily involved in extraoral digestion, which is necessary for consumption of prey, as suggested by their low but detectable expression in the venom glands and presence in the secreted venom but upregulation in the midgut. The second major cluster contains components that are more or less exclusively associated with venom—henceforth referred to as “core venom.” While the 2 venom glands share a similar gene expression profile, our venom-focused comparative analysis also revealed that MXVG and CVG form 2 distinct clusters by hierarchical clustering (Fig. 3c, top dendrogram). This distinction is due to the subclustering of the genes of the core venom genes (second major cluster; Fig. 3c, left dendrogram), where one subcluster is expressed in both CVG and MXVG, while the other subcluster is expressed more highly in the MXVG than in the CVG.

Next, we compared the candidate venom components identified in this study of *C. carnea* with those previously reported for the same species by Fischer et al. (2024). Specifically, we mapped 119 transcripts from the previous study to the *C. carnea* genome. These transcripts were selected based on proteomic support and the presence of a predicted signal peptide, including those with uncertain predictions marked with a question mark (Fischer et al. 2024). We mapped these transcripts to our *C. carnea* genome while allowing for multimapping. To avoid inflating gene counts, we consolidated noncontiguous mappings (incomplete transcripts mapping to the same gene) and multiple isoforms into single representative sequences per gene, yielding 97 putative genes. Of these 97 genes, 64 overlapped with our set of 277 venom genes (including 10 from the core venom set; SI Data S2), 31 were found in our *C. carnea* genome annotation, and 2 were not found in our annotation. A closer inspection of hemolysin-like transcripts identified in the *C. carnea* transcriptome by Fischer et al. (2024) revealed that they do not map to the *C. carnea* genome. Instead, they map to genes from predatory hemipteran families such as Nepidae, Belostomatidae, and Reduviidae, suggesting that they may represent contaminants (SI Table S3).

We then compared the venom of *C. carnea* with that of the European antlion (Euroleon nostras, Myrmeleontidae; Fischer et al. 2024). Using the same mapping strategy as above for the venom proteotranscriptome of *E. nostras* mapped against its own genome (Fischer et al. 2024), we found that the 252 transcripts from *E. nostras* collapsed into 193 genes. The *E. nostras* venom gene dataset was then compared to the 2 *C. carnea* venom gene datasets using OrthoFinder to re-examine the overlap in composition between *E. nostras* and *C. carnea*. This approach revealed that the genes in our *C. carnea* dataset recovered all orthogroups found in the previous *C. carnea* venom annotation (SI Table S4). Furthermore, comparison of our *C. carnea* venom-gene dataset with that of *E. nostras* identified only 13 orthogroups unique to the latter (SI Table S5). These results highlight the importance of using complementary methods for comprehensive venom characterization and suggest that the degree of compositional divergence between lacewings and antlions may be lower than previously assumed (Fischer et al. 2024), despite their divergence time of approximately 200 million years (Vasilikopoulos et al. 2020).

### The green lacewing secretes an enzyme-rich and diverse venom

Of our candidate venom components, enzymes constituted the largest fraction of genes with ∼42% (Fig. 3a) of the *C. carnea* venom genes. These enzymes include enzyme families convergently recruited into other venomous lineages, such as S1 serine proteases, several glycoside hydrolase families including chitinases (family 18), C1A cysteine proteases, and M10 metalloproteases, that are consistent with the extra-oral digestion feeding strategy of *C. carnea* larvae. Additionally, the venom of *C. carnea* is rich in genes encoding carbohydrate-binding proteins, such as those containing a ricin-type B-like lectin domain, which are also found in the venom of mosquitoes (Kern et al. 2021). We also identified apolipophorin-III-like proteins, which are typically associated with the venom of phytophagous insects (Bleau et al. 2024). However, the function of these proteins in lacewing venom remains uncertain.

We also identified a rich diversity of additional enzyme types that are probably not involved in extraoral digestion. Among these was a newly identified phospholipase A2 (PLA2) enzyme. These enzymes have been independently recruited into venoms across a wide range of animal lineages, likely because cell membranes are a general and highly accessible toxin target (Schendel et al. 2019). Aligning this PLA2 to those previously identified in the European antlion (Fischer et al. 2024) revealed that they are not toxin orthologs but likely represent independent recruitment events, as indicated by differences in the number of disulfide bonds and by the observation that one PLA2 identified in the European antlion is fused to apolipoprotein and lacks a signal peptide (SI Figure S3). We also identified type A1 phospholipase (PLA1; 1 gene in core venom, SI Figure S4), M2 metalloproteases (core venom), glycosyl hydrolase family 22 (GH22; core venom), glycosyl hydrolase family 27 (GH27; shared venom), and carboxylesterase type B (paralogs in both shared and core venoms). PLA1 is a major component of several hymenopteran venoms (Perez-Riverol et al. 2019), while carboxylesterase type B is a major component of centipede venoms (Undheim et al. 2014).

As observed by SDS–PAGE, *C. carnea* venom also contains a large number of nonenzymatic high-molecular-weight proteins (Fig. 3b). Among these large proteins, we identified large amounts of vitellogenin-like proteins, which have been found in the venom of bees (Blank et al. 2013) and phloem-feeding insects (Bleau et al. 2024), and an Rhs-like protein similar to one of the major components of mosquito venom (King et al. 2011; Liu et al. 2023). In addition, we found leucine-rich repeat-containing proteins (LRRP) in the venom, a gene family that has been shown to be expanded in another green lacewing species (Wang et al. 2022) and was also reported in the previous venom proteome analysis of the *C. carnea* (Fischer et al. 2024). LRRP's have also been identified in the venom of the gall-forming Hessian fly larvae (*Mayetiola destructor*; Diptera: Cecidomyiidae), where they are thought to defeat immunity and alter growth of the host plant (Zhao et al. 2015). We also identified 7 genes in the core venom that encoded proteins without similarity to any previously characterized proteins or domains, which we therefore labeled as uncharacterized. Interestingly, several of these proteins had high predicted mean disorder (SI Figure S5), which is intriguing as such regions can have context-specific functions (Holehouse and Kragelund 2024), making them potentially interesting venom components.

In addition to enzymes and larger nonenzymatic proteins, we found 8 families of peptides that have been convergently recruited into other venoms. These peptides included protease inhibitors such as Kazal (Yang et al. 2020), pacifastin (Ruder et al. 2013; Qian et al. 2017; Walker et al. 2019), and trypsin inhibitor-like (TIL) peptides (Zeng et al. 2002; Michel et al. 2012; Chen et al. 2013, 2015) that could function to inhibit protease activity of venom enzymes prior to secretion, target prey proteases or nonprotease receptors, or both (Mourão and Schwartz 2013; Chen et al. 2015). We also identified peptide families previously shown to have antimicrobial activity, such as attacins (Buonocore et al. 2021) and the extremely widespread cystine-stabilized alpha–beta-type defensins (CSab) (Dash et al. 2019). Furthermore, we identified von Willebrand factor type C-like (SVWC) peptides, which are common in arthropod venoms (Undheim et al. 2014; Drukewitz et al. 2018; Reichhardt et al. 2020; Gong et al. 2022). Among the detected venom SVWC were both variants that displayed the conserved cysteine conformation typical of arthropod SVWCs and a variant with an extra disulfide bond that diverged from the usual 4 disulfide bridges found in arthropod SVWCs (SI Figure S6c).

Interestingly, the richest of the identified peptide families were odorant-binding peptides (OBPs), of which we found 15 paralogs encoding peptides identified in the venom proteome. These disulfide-rich peptides are typically associated with chemoreception (Sun et al. 2018; Rihani et al. 2021), but have also been identified in venom proteomes of lepidopteran larvae (Moneo et al. 2003; Seldeslachts et al. 2024) and parasitic wasps (Wang et al. 2015). Twelve of the venom odorant-binding proteins (OBPs) belong to a novel 6-cysteine scaffold representing a noncanonical fold distinct from other known 6-cysteine OBPs (Hekmat-Scafe et al. 2002) and are arranged in a tandem gene array (SI Figure S7). We designate these as N-OBPs (Neuroptera OBPs). Structural modifications such as alterations in cysteine frameworks and residue spacing are indicators of functional innovation (Undheim et al. 2015; Pineda et al. 2020), suggesting these newly identified OBP variants may have acquired new venom-specific functions. Interestingly, 10 of these N-OBPs belong to the core venom. Full-length nonchimeric read (FLNC) counts for the N-OBPs detected in our PacBio Iso-Seq reads also indicated that most venom N-OBPs were primarily detected in larvae, except for N-OBP9 and N-OBP19, which were detected in both developmental stages, and N-OBP16, which was adult-specific (SI Figure S8). However, N-OBP16 is part of the core venom, and thus, the lack of Iso-Seq reads from larvae is unexpected and possibly due to technical limitations such as lower coverage of shorter transcripts in this method. Finally, we identified 11 genes encoding peptides with hits against cuticle protein annotations, with 5 belonging to the core venom. FLNC counts indicated that 2 cuticle proteins were mostly expressed in larval stages, with 1 sharing 50% of counts with adults + eggs. However, whether these cuticle proteins represent functional venom components or contamination from the cuticularized venom delivery structures remains unclear and should be further investigated.

### Green lacewing venom components both originate from gene duplication and gene co-option

**Figure 4:**
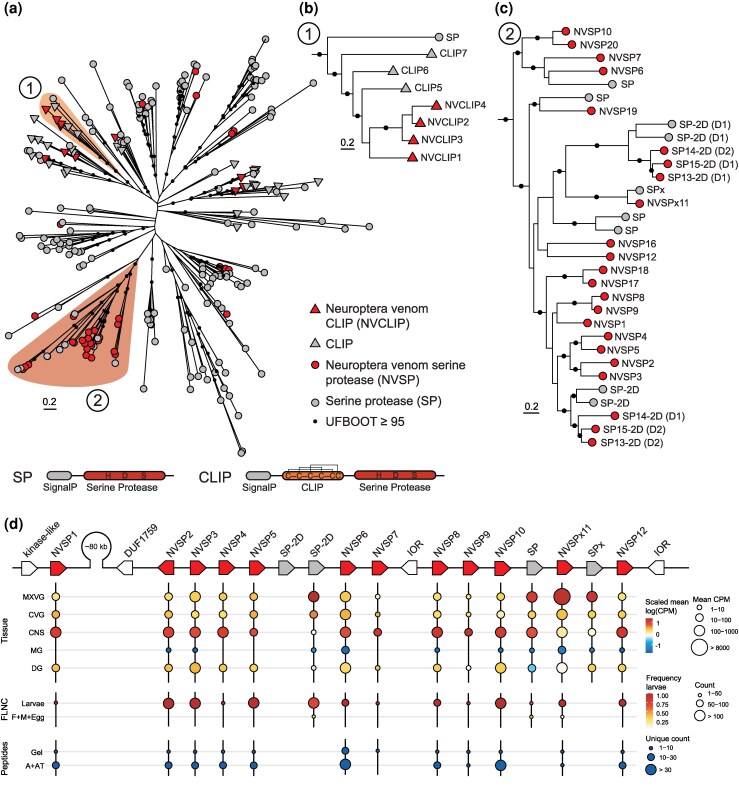
Evolution, expression, and genomic architecture of 2 functional classes of serine proteases in *C. carnea* venom. a) Domain architecture of 2 major serine protease classes: one containing only the canonical trypsin-like serine protease domain (SP; bottom) and the other class containing an additional CLIP domain (top). An unrooted phylogeny of all trypsin-domain-containing serine proteases identified in the genome reveals 2 primary clades—one enriched for CLIP-domain proteins and the other for SPs. Proteins identified in the proteome are labeled Neuropteran venom serine proteases (NVSPs) or Neuroptera venom CLIP proteases (NVCLIPs). The phylogeny tree file is available as SI Data S3. b) Extracted subtree from clade marked 1 showing the phylogenetic placement of NVCLIPs. c) Extracted subtree of NVSPs from clade marked 2, highlighting a lineage-specific expansion. Bootstrap support values ≥ 95 (UFBoot) are indicated with black circles. d) Genomic organization, tissue-specific expression, and peptide-level proteomic support for NVSP genes. The NVSPs form a tandem gene array and exhibit varying expression levels across tissues, but they are mainly expressed during larval stages (FLNC). The gene expression (top) is shown as a color-scaled mean log (CPM) across tissues, with a dot size that corresponds to the mean CPM. For expression across life stages (FLNC), color reflects the frequency of reads within larval samples, while size represents the number of reads counted. Proteomic support (bottom) is shown by detected peptides, with dot size indicating the number of unique peptides identified.

Among the most abundant components in the venom of *C. carnea* are venom S1 serine proteases, which are found in a wide range of animal venoms. We identified 46 genes encoding proteins with S1 and signal peptide domains that were found in the venom proteome (Fig. 2). A search of the *C. carnea* genome annotation for trypsin domains revealed 227 putative paralogs with S1 domains, indicating a massive expansion of this gene family. Phylogenetic analysis of these paralogs shows that they cluster into one group (Fig. 4a) with S1 domain/s and another group enriched with S1 genes with an additional N-terminal CLIP domain, which is a regulatory domain that, among others, plays important roles in the innate immune systems in insects (Kanost and Jiang 2015). We refer to the venom-identified CLIP-domain serine proteases as NVCLIPs (Neuroptera Venom CLIPs) and the other venom-identified serine proteases as NVSPs (Neuroptera Venom Serine Proteases). Our phylogenetic analysis also suggests that NVCLIPs and NVSPs have both been recruited to the venom on multiple occasions (Fig. 4).

Among the NVCLIPs, we found paralogs upregulated in venom-producing tissues (Fig. 4, SI Figure S9) and paralogs with larval-specific expression, indicating that these are indeed venom-specific genes. Of the 11 NVCLIPs identified in the venom, 5 NVCLIPs are highly upregulated in venom-producing tissue (i.e. part of the core venom), 3 of which are contained in a clade containing only paralogs detected in the venom in our phylogenetic analysis of the S1 gene family (Fig. 4b). The fourth paralog contained in this clade (Fig. 4b), *NVCLIP4*, showed very low expression in all tissues but was still detected in the venom, while 1 of the 3 highly expressed NVCLIPs was also detected in the nonvenomous adult stage (SI Figure 9, *NVCLIP2*). The remaining 6 NVCLIPs were upregulated in venom-producing tissue but also in at least one other tissue. This indicates that some NVCLIPs have evolved via gene duplication of venom-specific paralogs, while other NVCLIPs have most likely retained their ancestral function while also being co-opted for a function in the venom (SI Figure 9). CLIP-domain serine proteases are also found in the venom of the endoparasitoid wasp *Cotesia rubecula,* where they were shown to suppress the host immune melanization response (Zhang et al. 2004). However, given the rapid melanization in *D. melanogaster* larvae upon injection of venom either by or from *C. carnea* suggest that is not the case for NVCLIPs, whose activity and function in the venom remain unknown.

Among the NVSPs, one gene array stood out as containing a particularly high number of paralogs, including paralogs encoding some of the most abundant NVSPs identified in the SDS-PAGE analysis, several of which were expressed in both venom-producing tissues as well as in the CNS and DG (Fig. 6d). This expression profile suggests that these serine proteases may serve multiple functions across tissues in addition to their role in venom. Interestingly, one serine protease, encoded by *NVSPx11*, lacked the catalytic triad and was differentially upregulated in venom-producing tissues, indicating that it may have undergone neofunctionalization similar to the nonenzymatic venom PLA_2_ in certain viperid snakes (Lomonte and Rangel 2012). Phylogenetic analysis placed *NVSPx11* within a group of double-domain serine proteases, one of which also lacked the catalytic triad. This suggests that *NVSPx11* may have evolved through gene duplication and domain loss. Although the functions of NVSPs remain unknown, they likely serve multiple roles that include, but are not limited to, extra-oral digestion, as indicated by the existence of distinct classes and the presence of loss-of-function mutations.

Taken together, these results show that the gene families encoding *C. carnea* venom components have complex evolutionary histories that include a mix of co-option, recruitment following duplication, diversification of venom-paralogs by gene duplication, and functional innovation of new paralogs through both small structural and large architectural changes.

### Green lacewing venom provides insight into the emergence of novel molecular traits

**Figure 5:**
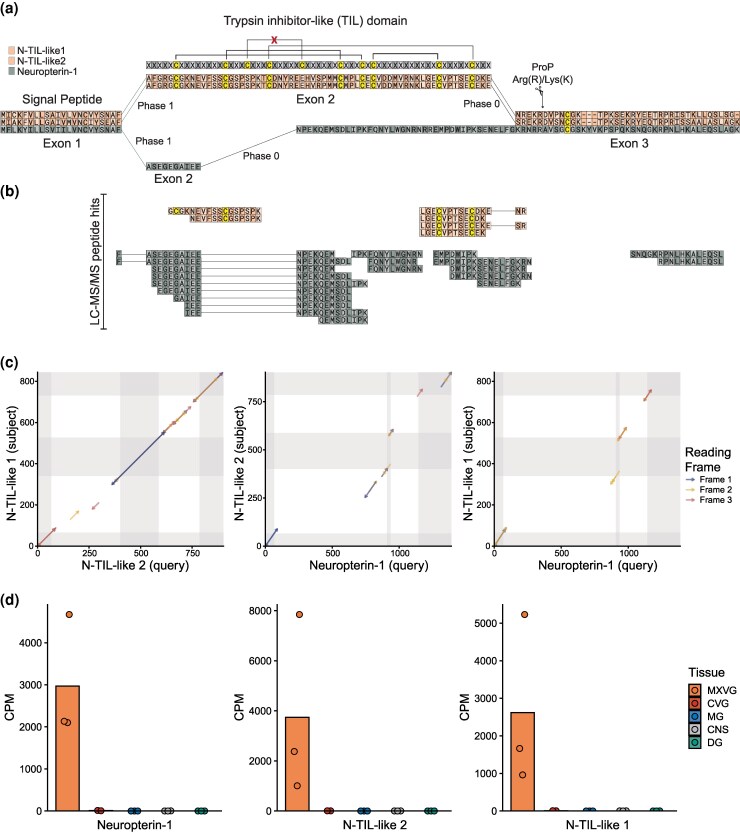
Venom peptide evolution through gene duplication and domain loss. a) Multiple sequence alignment of 2 trypsin inhibitor-like (TIL) domain peptides and a derived paralog showing maintained exon structure, signal peptides, and intron phases. b) Unique peptide mapping via LC–MS/MS peptide hits across the protein sequence domains. c) TblastX sequence similarity plots (dot plots) between the 3 paralogs, including intronic sequences, showing conserved regions across different reading frames highlighted in gray. d) Tissue-specific expression profiles of the 3 gene variants across different tissue types.

To examine whether the uncharacterized components in the core venom that showed no similarity to previously characterized proteins or domains could be divergent forms of other *C. carnea* venom components, we performed a BLASTP search of their full coding sequences against all other core venom genes (e-value cutoff: 1e-5). Surprisingly, one of these uncharacterized peptides that lacked cysteines showed sequence similarity to 2 genes that share a cysteine framework with trypsin inhibitor-like (TIL) domains. These domains are annotated as TIL domains but lack the canonical 3 to 5 disulfide bonds. We refer to these as N-TIL-like-1 and N-TIL-like-2 (neuroptera trypsin-inhibitor-like). Although the overall sequence similarity between the peptide and the N-TIL-like genes was low (Fig. 5a and c), all 3 genes present a similar protein pattern consisting of a similar signal peptide, exon-intron structure, and predicted cleavage site. These features suggest that they originated from a common TIL-like ancestor through gene duplication events. LC–MS/MS analysis of milked venom revealed a putative pattern of post-translational processing (Fig. 5b). The 2 N-TIL-like paralogs showed no peptide hits corresponding to their C-terminal domains, suggesting cleavage of this region before secretion. The uncharacterized peptide shared this cleavage site with peptide hits for this region identified in only 1 of 3 venom samples, whereas the N-terminal region yielded numerous peptide hits (Fig. 5b). Given its high expression, presence in the venom, and likely neofunctionalization, we designate this protein as “Neuropterin-1.” This pattern suggests that the N-terminal region following the signal peptide is the active component of the putative toxin.

Cysteine-rich peptides typically maintain structural robustness through disulfide bonds. These bonds allow mutations to accumulate in nonstructural residues without compromising overall peptide integrity, thereby enabling functional diversification, a pattern often seen in cysteine rich toxin peptide families (Undheim et al. 2016). In contrast, Neuropterin-1 has completely lost its cysteine-rich TIL domain. One possible explanation is that the retention of the C-terminal region still provided a functional phenotype after losing the TIL domain following gene duplication, allowing the N-terminal cysteine-free domain to evolve freely or acquire a novel function. Thus, Neuropterin-1 represents a compelling example of how gene duplication, domain loss, and modular evolution can contribute to the emergence of novel toxins and highlights the usefulness of integrating genomic data in unraveling their evolutionary origins. While domain-loss has previously been suggested to have been a crucial innovation in the evolution of three-finger toxins in snakes (Koludarov et al. 2023), the retained ancestral domain of Neuropterin-1 appears to not be a crucial functional component, based on its likely low abundance in the venom. Future work should examine the functions of both the N- and C-terminal regions of Neuropterin-1 to test the hypothesis of neofunctionalization.

### Alternative splicing is an important contributor to the venom arsenal of the green lacewing

**Figure 6:**
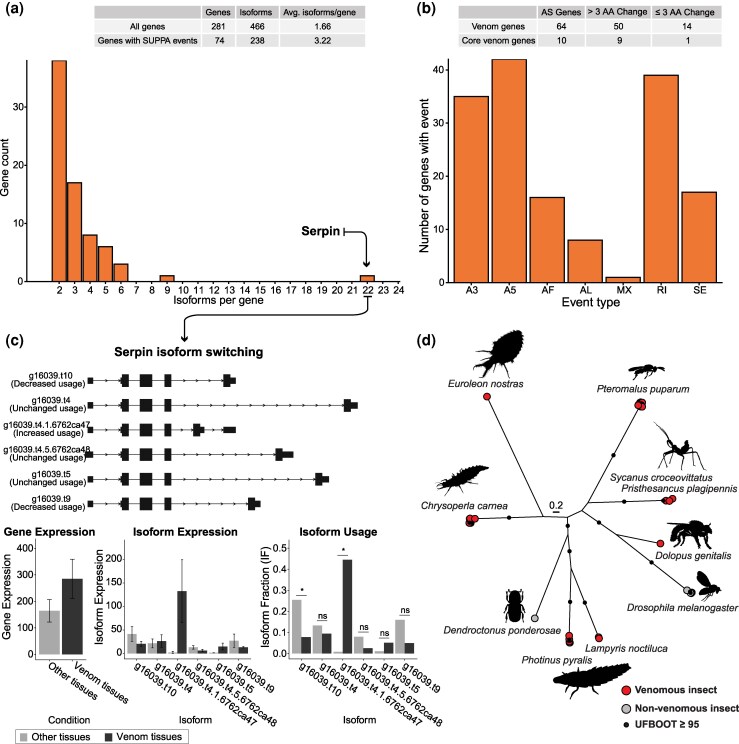
Alternative splicing patterns in *C. carnea* venom and convergent recruitment of splicing gene in venomous insects. a) Number of alternatively spliced genes identified by SUPPA2 generateEvents function and corresponding isoform counts per gene. Inset table shows a comparison of isoforms per gene between the total venom gene count and SUPPA2-identified alternatively spliced genes. b) Distribution of alternative splicing event types per gene identified by SUPPA2. Splicing events include skipped exons (SE), mutually exclusive exons (MX), alternative 5′ splice sites (A5), alternative 3′ splice sites (A3), retained introns (RI), alternative first exons (AF), and alternative last exons (AL). Table below summarizes alternative splicing analysis of venom genes and core venom genes (differentially expressed in venom tissues), categorized by predicted isoform sequence changes (indels ≤3 or >3 consecutive amino acids). c) Isoform switching analysis of Serpin using IsoformSwitchAnalyzeR, showing switching isoforms with corresponding gene expression (left), isoform expression (center), and isoform usage (right). Increased and decreased usage refer to higher or lower, respectively, expression of a splice variant relative to the expression of other splice variants in venom-producing tissue. d) Maximum likelihood phylogenetic tree of Serpin orthologs from selected venomous insect lineages, demonstrating independent recruitment of an alternative spliced gene across venomous taxa. The phylogeny tree file is available as SI Data S4. The image used for *D. ponderosae* was *Ips typographus* by Dorota Paczesniak, shared under CC by 4.0 (https://creativecommons.org/licenses/by/4.0/).

While toxin gene family expansions represent the most common form of toxin arsenal diversification, we also observed that alternative splicing events are relevant for the venom composition of the green lacewing. However, although alternative splicing has been observed in some snakes and a wasp (Ogawa et al. 2019; Gopalan et al. 2022; Pan et al. 2023; Ye et al. 2023), the prevalence of alternative splicing of toxin genes in venomous animals remains poorly known. Leveraging our high-quality genomic and long-read transcriptomic datasets, we examined the source of molecular diversity for each venom gene. Using SUPPA2 to categorize local splicing events among the 466 isoforms distributed across 281 genes, we identified alternative splicing events in 74 genes, accounting for 183 isoforms (Fig. 6a). To determine which alternative splicing events resulted in functionally relevant changes to protein diversity, we identified putative venom genes with indels of more than 3 consecutive amino acid changes between isoforms. Among the core venom genes, we found that 9 of the 10 genes exhibiting alternative splicing also showed amino acid variation (>3 AA changes, Fig. 6b, SI igure S10), suggesting that alternative splicing can generate functionally distinct protein variants in *C. carnea*. However, 14 of the 64 putative venom genes did not result in differences in protein-coding regions, suggesting these may instead be related to regulation of toxin expression. Exploring these patterns further, for instance through single-cell RNA sequencing, could uncover cell-type-specific expression and fine-scale regulatory processes similar to those described for different toxin paralogs in, e.g. snake venom glands (Nachtigall et al. 2025a).

We next examined whether alternative splicing provides a means of reducing evolutionary conflict in genes with both venomous and nonvenomous functions by testing for differential usage of splice isoforms between venom and nonvenom tissues. We found that many of the serpin isoforms identified in the venom are encoded by a single, alternatively spliced serpin gene (core venom) whose splice variants differ in relative expression between tissues (Fig. 6c). Splice variants of this serpin gene have previously been studied in the parasitoid wasp *Pteromalus puparum* (Yan et al. 2023; Ye et al. 2023), where *PpSerpin-1* exhibits exon duplications that enable alternative splicing of the exons to generate functional diversity (Yan et al. 2023). These isoforms serve dual roles in the wasp's immunity and as venom components across larval and adult stages, with specific variants inhibiting host prophenoloxidase activation and suppressing host melanization (Yan et al. 2017, 2023). These activities are similar to that of a serpin from the venom of the assassin bug *Sycanus croceovittatus*, which was also demonstrated to have a dose-dependent insecticidal effect on the army worm *Tenebrio molitor* (Liang et al. 2025). Similarly, we observed that this serpin gene is expressed across various tissues (Fig. 6d) and throughout different life stages (SI Figure S8) in *C. carnea*. Surveying for this serpin gene across other venomous insect lineages, including robber flies (Walker et al. 2018a), assassin bugs (Walker et al. 2017), and the firefly *Lampyris noctiluca* (Krämer et al. 2024), reveals that orthologs of this gene have been independently recruited into the venom of multiple venomous insects. This pattern suggests that alternative splicing may be a widespread mechanism for enhancing protein diversity in venomous insect lineages, primarily by expanding toxin arsenals while reducing evolutionary conflict in genes that perform both physiological and venomous functions, such as in the case of snake venom phosphodiesterase (Pan et al. 2023).

## Conclusion

Using a suite of methods, we demonstrate that—contrary to previous suggestion—the venom of *C. carnea* is both cytotoxic and has paralytic activity against model prey and is produced by a well-developed venom system comprising 2 pairs of venom glands that is not connected to the digestive system. A comprehensive comparative venom composition analyses also reveal that the venom of *C. carnea* is far more complex than previously recognized, containing multiple enzymatic and nonenzymatic—as well as several completely uncharacterized—proteins and peptide families. We demonstrate that multiple molecular evolutionary mechanisms have shaped this venom arsenal, including gene duplication, neofunctionalization, domain loss, and co-option of existing genes across tissues and venomous and nonvenomous life stages. Beyond these “classical” mechanisms of venom evolution, our findings identify alternative splicing as a contributor to the toxin arsenal of *C. carnea*. The alternative splicing observed seems to facilitate both the co-option of genes for novel venom functions and the generation of additional toxin isoforms that expand the toxin diversity of the venom. In addition, our findings suggest that alternative splicing may also distribute the expression and secretion of abundant toxins across different populations of toxin-secreting cells within venom-producing tissues. Taken together, our results provide a comprehensive overview of the evolutionary genetic mechanisms shaping a molecular adaptive trait and advance our understanding of venom system function and evolution in insects.

## Materials and methods

### Sample acquisition and venom collection

Commercially available *C. carnea* were purchased from Borregaard Bioplant. From the acquired animals, we select a female and a male for crossing. The male (BM3) was used for genome sequencing as it is the heterogametic sex. Its offspring were used to generate life-stage-specific long-read transcriptomes and proteomic characterization of venom composition that was used for genome annotation and splice variant identification. In addition, we collected *C. carnea* from Blindern, Oslo, Norway, and reared them as a population in the lab. The Oslo individuals were used for venom system morphology, tissue-specific gene expression, and additional venom extraction for SDS–PAGE and biological assays.

We developed a venom extraction protocol that allows for multiple extractions from individual animals. The method involves slowly applying CO_2_ to immobilized larvae and was developed after observations that larvae anesthetized with CO_2_ prior to dissection often secreted venom just prior to and while recovering from becoming immobilized. Third-instar larvae were starved for 24 h before extraction. Individual larvae were then gently positioned using soft biological forceps and placed under a ZEISS Stemi 508 stereo microscope. Venom release was induced by carefully administering CO₂ directly to the larva (SI Video S1). Each venom sample was collected using a gel-loading pipette tip and immediately suspended in 5 µL of ice-cold Milli-Q water and kept on ice until it was snap frozen and stored at −80 °C.

### Genome sequencing and assembly

High-molecular-weight DNA was extracted from the whole body tissue of one male *C. carnea*, referred to as BM3, using the standard Qiagen MagAttract HMW kit protocol. HiFi sequencing libraries for BM3 were prepared using the Pacific Biosciences protocols for low DNA input using the SMRTbell Express Template prep kit 2.0, with the exception of nuclease treatment during the library step. DNA was fragmented using Megarupter 3. The library was sequenced on one 8 M SMRT cell on a Sequel II instrument using Sequel II Binding kit 2.0 and sequencing chemistry v2.0. Loading was performed by diffusion, with pre-extension for 2 h and a movie time of 30 h. CCS sequences were generated using the CCS pipeline (SMRT Link v9.0.0.92188) with a minimum number of passes of 1 and a minimum predicted accuracy of 0.99. Cutadapt v3.4 (Martin 2011) was used to remove any remaining adapter sequences before genome assembly was done using Hifiasm v0.15-r327 (Cheng et al. 2021). The resulting assemblies were assessed using BlobToolKit (Challis et al. 2020) and BUSCO v5.5.0 (Manni et al. 2021) with endopterygota_odb10 (2,124 total number of genes) as the database.

### Iso-seq transcriptome sequencing and assembly

RNA from offspring of BM3 was extracted from 5 eggs, 4 first-instar larvae (L1), 1 second-instar larva (L2), 1 third-instar larva (L3), and 1 each of imago male and female using the standard Qiagen RNeasy-mini kit protocol. The library for sequencing was prepared using the Pacific Biosciences protocol for Iso-Seq Express template preparation for Sequel and Sequel II systems. Samples were barcoded during cDNA amplification and pooled equimolarly during library prep. The library was sequenced on a Pacific Biosciences Sequel II instrument using Sequel II Binding kit 2.0 and Sequencing chemistry v2.0. Sequencing was performed on one SMRT cell. Loading was performed by diffusion. Movie time was 24 h, with a pre-extension time of 2 h. CCS reads were obtained with SMRT Link v9.0.0.92188 using a minimum number of passes 3 and a minimum accuracy of 0.99. HiFi reads were further analyzed using the Iso-Seq pipeline v4.0.0. Primer removal and demultiplexing were performed using lima v2.9.0. Demultiplexed reads were trimmed of poly(A) tails and concatemers were removed using the isoseq refine function with default settings, followed by clustering using isoseq cluster with –use-qvs. The clustered HiFi reads were mapped to the BM3 genome using the pbbm2 v1.11.99 preset for Iso-Seq reads. The mapped reads were collapsed using isoseq collapse with default settings. Full-length nonchimeric read (FLNC) counts were summed across annotated genes (see Genome annotation section below) to obtain read counts across life stages.

### Tissue dissection and RNA-sequencing

L3 larvae were selected from the Oslo population for tissue dissection after being switched from a diet of moth eggs to a *Drosophila* diet 24 h before dissection. After a starvation period of 6 to 8 h, the larvae were euthanized using CO₂ gas and secured with minute pins to a SYLGARD 184-coated dissection plate (Dow Inc.). Dissections were performed under a ZEISS Stemi 508 stereo microscope in Milli-Q water, using Dumont No. 5CO forceps and a 30G needle to carefully isolate the desired tissues. Immediately after dissection, the extracted tissues were snap-frozen and kept at −80 °C until further use. The dissected tissues included the cephalic venom gland (CVG), maxillary stylets (MX), which include the lateral and medial venom glands (Fischer et al. 2024), central nervous system (CNS) including the brain and subsequent ganglia, foregut (FG), midgut (MG), and defense glands (DG; anal glands that produce a defensive secretion). To obtain sufficient RNA yields for each tissue type, tissues dissected from 3 sets of 5 individuals (15 animals in total) were pooled to generate a total of 3 biological replicates for each tissue type. Total RNA was extracted from the different tissues using TRIzol Reagent (Thermo Fisher), following the manufacturer's protocol except for the addition of an overnight precipitation step after the addition of 1% RNA-grade glycogen (Thermo Fisher). Contamination levels and RNA concentrations were measured using a NanoDrop 1000 spectrophotometer (Thermo Fisher), and RNA integrity was assessed with the Agilent 2100 Bioanalyzer pico kit (Agilent Technologies).

Tissue-specific libraries were prepared using the Illumina TruSeq stranded mRNA kit with 100 ng of input RNA per sample (except for sample 15 with 50 ng input and sample 25 with 42 ng input), following the manufacturer's protocol. Sequencing was performed on a NovaSeq X instrument (Illumina) using one lane of a 25B flow cell, employing 150 bp paired-end reads according to the manufacturer's recommendations. Base calling was performed on the NovaSeq X instrument using software version 1.2.2.48004. The data were then converted to FASTQ format and demultiplexed using BCL Convert v4.1.23. Resulting reads were trimmed with Trim Galore v0.6.10 (Krueger et al. 2023) using CutAdapt 4.2 (Martin 2011), and quality control was performed using FastQC v0.11.9.

For annotation purposes, we also sequenced RNA extracted from the whole body of an L3 larva using the standard Qiagen RNeasy mini-kit. The extracted RNA was prepared and sequenced as per the tissue-specific samples.

### Genome annotation

To build a comprehensive set of proteins for downstream annotation, we combined several methods. First, reads from all tissues and the whole-body transcriptome were mapped to the BM3 genome using HISAT2 v2.2.1 (Kim et al. 2019), and the resulting alignments were sorted with SAMtools v1.16.1 (Danecek et al. 2021). These BAM files were initially used for genome-guided assembly with Trinity v2.15.1 (Grabherr et al. 2011; Haas et al. 2013). Subsequently, the BAM files were filtered for properly paired reads (-f 2), and the filtered reads were extracted using BEDtools v2.30.0 (Quinlan and Hall 2010) bamtofastq. These extracted reads were then used for de novo assembly with Trinity. Additionally, StringTie v2.2.1 (Pertea et al. 2015) was employed to assemble transcripts for each tissue, and these assemblies were merged using the stringtie merge utility. Replicates of the same tissue were assembled together, while the whole-body transcriptome was assembled separately. PASA v2.5.2 (Haas et al. 2003). PASA_comprehensive_db was used to integrate a concatenated set of all assemblies, incorporating the Iso-Seq-assembled transcriptome for increased completeness. The resulting transcriptome was translated into protein sequences using CodAn v1.2 (Nachtigall et al. 2021) using the INV_full model and combined with OrthoDB v11 (Kuznetsov et al. 2023), which served as input for BRAKER3 v3.0.8 (Gabriel et al. 2024) along with the BAM files produced by the mapping with HISAT2 (above). Finally, we used PASA v2.5.2 to align the Iso-Seq data and update the annotation to include exon boundaries and untranslated regions and then the script agat_sp_fix_cds_phases.pl from AGAT (Dainat et al. 2024) was used to fix the CDS phases. Annotation completeness was assessed using BUSCO v5.5.0 with the database endopterygota_odb10.

### Proteomic analyses

Extracted venom from individual L3 larvae was analyzed in triplicate using a shotgun approach. Venom was diluted to approximately 0.5 µg/µL in 50 mM ammonium bicarbonate (ABC) and cysteines were reduced in 5 mM dithiothreitol (Sigma Aldrich) and alkylated with 10 mM iodoacetamide (Sigma Aldrich). One aliquot of the reduced and alkylated venom was processed without further treatment (A samples in SI Data S1). The second aliquot (AT samples in SI Data S1) of the reduced and alkylated venom was digested with sequencing-grade trypsin (Sigma Aldrich) at a 1:50 enzyme:substrate ratio by incubating for 1 h at 37 °C and then 4 min microwave treatment at lowest power before the reaction was stopped by adding formic acid (FA) to a final concentration of 5% (vol/vol). The tryptic peptides were desalted using C18 ZipTips (Thermo Fisher), dried in a vacuum centrifuge, and reconstituted in 5% acetonitrile (ACN) and 0.5% FA (vol/vol).

The desalted digested samples were analyzed by LC–MS/MS using a Q Exactive Fourier transform mass spectrometer (Thermo Fisher, USA) coupled to an Ultimate 3000 nano-UHPLC system (Dionex, USA). Peptides were separated on an Acclaim PepMap 100 column (C18, 3 µm particles, 100 Å poresize, 75 μm inner diameter) (Dionex, USA) using a flow rate of 300 nL/min and a gradient of 3% to 80% solvent B (90% ACN, 0.1% FA) in 0.1% FA over 27 min. Survey MS spectra were acquired across 400 to 2,000 *m*/*z* at a resolution of 70,000 at 200 m/z. Higher-energy collisional dissociation (HCD) MS2 scans were performed at 200 to 2,000 *m*/*z* at a resolution of 17,000 using an isolation width of 2.0 *m*/*z*, a threshold of 10^5^ ion counts and a maximum accumulation time of 60 ms.

For SDS–PAGE, we used NuPAGE 4% to 12% Bis–Tris Mini Protein Gels (Thermo Fisher) following the manufacturer's guidelines (Pub. No. MAN0007891, Rev. D) with samples reduced using NuPAGE Reducing Agent (10×) and the Mark 12 Unstained Standard (2.5 to 200 kda range) as our ladder. The gel was stained overnight in Blue Silver (Candiano et al. 2004) and destained with 1% acetic acid. Visible bands were excised from the gel and subjected to in-gel digestion. For the digestion, gel pieces were washed with Milli-Q water for 10 to 30 min, then destained with 50% ACN in 40 mM ABC for 20 to 60 min. Gel pieces were dehydrated with ACN until opaque, then rehydrated with 20 ng/µL sequencing-grade trypsin (Sigma Aldrich) for 20 min on ice before overnight incubation at 37 °C. The reaction was stopped with FA to a final concentration of 1% (vol/vol). Peptides were extracted twice with 5% FA/50% ACN and then 5% FA/90% ACN. Extracts were dried in a vacuum centrifuge, reconstituted in 5% ACN and 0.5% FA, desalted using C18 ZipTips (Thermo Fisher), dried in a vacuum centrifuge, and reconstituted in 5% acetonitrile (ACN) and 0.5% FA prior to analysis by LC–MS/MS.

The samples were analyzed by LC–MS/MS using a timsTOF Pro (Bruker Daltonik, Bremen, Germany) coupled to a nanoElute nanoflow liquid chromatography system (Bruker Daltonik, Bremen, Germany) via a CaptiveSpray nanoelectrospray ion source. The peptides were separated on a reversed phase C18 column (25 cm, 75 µm inner diameter, 1.5 µm particle size, 100Å pore size, PepSep, Marslev, Denmark) heated to 50 °C. The peptides were separated by a gradient from 0% to 35% solvent B (90% ACN, 0.1% FA) in 0.1% FA over 30 min at a flow rate of 300 nL/min. MS acquisition was performed in DDA-PASEF mode. The capillary voltage was set to 1.5 kV with a mass range of 100 to 1,700 *m*/*z*. The number of PASEF ranges was set to 20 with a total cycle time of 1.16 s, charge up to 5, target intensity of 20,000, intensity threshold of 1,750, and active exclusion with release after 0.4 min. An inversed reduced TIMS mobility (1/k0) of 0.85 to 1.40 Vs/cm^2^ was used with a range time of 100 ms, an accumulation time of 100 ms, a duty cycle of 100%, and a ramp rate of 9.51 Hz. Precursors for data-dependent acquisition were fragmented with an ion mobility-dependent collision energy, which was linearly increased from 20 to 59 eV.

The whole venom (Orbitrap-MS) and in-gel digest (tims-Q-TOF-MS) LC–MS/MS raw files were searched separately using MaxQuant v2.4.0.0 (Cox and Mann 2008) against our *C. carnea* annotation. For whole venom samples, 2 parameter groups were applied with unspecific digestion with no enzyme and 0 missed cleavages for A samples, and trypsin/P digestion allowing up to 2 missed cleavages for AT samples. Carbamidomethyl (C) was set as a fixed modification. For in-gel digests, Trypsin/P specificity was applied, allowing up to 2 missed cleavages, but no fixed modifications were set. For both sample types, variable modifications included oxidation (M), Glu->pyro-Glu, amidated (Protein C-term), and acetyl (Protein N-term). The precursor mass tolerance was set to 20 ppm for the first search and 4.5 ppm for the main search, and the fragment mass tolerance was 20 ppm. Peptide and protein FDR thresholds were set to 1%. Match-between-runs was disabled, and decoy sequences and a standard list of common contaminants were included automatically using MaxQuant's default settings.

### Micro-computed tomography and 3d-visualization

Specimens of *C. carnea* were fixated in alcoholic Bouin's solution (Duboscq-Brasil Fluid; Romeis 1989; Sombke et al. 2015) and subsequently stored in 70% ethanol (vol/vol). Prior CT scanning, the samples were dehydrated in an ascending ethanol series (15 min: 70%, 80%, 90%, 2 × 95% and 30 min: 2 × 99%, vol/vol), transferred to a 2% iodine solution (iodine, resublimated in 99% ethanol) for 12 h on a shaker and subsequently dried using a fully automatic Leica EM CPD300 critical point dryer (Leica, Wetzlar, Germany). µCT scans were performed using a Zeiss XradiaXCT-200 (Carl Zeiss AG, Oberkochen, Germany), using the 4× and 20× objective lens units with the following settings: 40 kV, 8 W, 200 mA, and pixel sizes of 2.6 µm and 0.6 µm for the 4× and 20× scans, respectively. For reconstruction the XMReconstructor software (Zeiss) was used, resulting in image stacks (TIFF format). Segmentation and volume rendering were performed using AMIRA v2024.2 (Thermo Fisher).

### Biological assays

The protein content of *C. carnea* venom was determined using the Qubit protein assay kit (Invitrogen) with a Qubit 2.0 Fluorometer, following the manufacturer's instructions.

All cell culture reagents used were from Thermo Fisher unless specified. *Drosophila* S2 cells were donated by Kaisa Louise Ingeborg Haglund (Oslo University Hospital, Norway) and maintained in Schneider's *Drosophila* media supplemented with 10 heat-inactivated fetal bovine serum (FBS) and 50 U/mL penicillin and 50 µg/mL streptomycin (growth media) at 28 °C without CO_2_ and were passaged every 2 to 3 d. Cell viability was evaluated using the CellTiter-Glo 2.0 Assay (Promega), an ATP assay. In 96-well plates (Greiner 655088), 30,000 cells per well were seeded and incubated overnight.

*C. carnea* venom and Triton X were prepared at 10× of the final concentration in growth media. Ten microliters of the venom and the controls were added to the appropriate wells and incubated for 24 h at 26 °C. The media was changed before adding 10 µL of CellTiter Glo 2.0 reagent. Plates were incubated in the dark for 10 min at 26 °C and luminescence was read using a Hidex Sense microplate reader (Hidex) with each well integrated for 1 s. To visualize dead cells, 10 µL of propidium iodide (Miltenyi Biotec) was added to each treated well. Brightfield and fluorescent (red channel) images were captured using a ZOE Fluorescent Cell Imager (Bio-Rad).

The paralytic effect of the venom was evaluated by intrathoracic injection of virgin male adult *D. melanogaster* (*n* = 9 to 12 flies per group). Microneedles were prepared by pulling glass capillaries (Drummond, 3-000-203-G/X) using a Sutter Instrument P-97 micropipette puller. The needle tip was trimmed with scissors, and the pipette was filled with mineral oil (Sigma) before attaching it to the Nanoinject II (Drummond). Flies were immobilized on a cold block on ice and were injected with 55.2 nL of the sample, with water as the negative control. After injection, the flies were transferred to a tube for observation. Paralysis was assessed using the righting and the climbing assays, expressed as *P*_0.5min_ and *P*_5min_, respectively. *P*_0.5min_ is the percentage of flies that right themselves within 0.5 min after injection, while *P*_5min_ denotes the percentage of flies that climb more than 3 cm from the bottom after tapping at 5 min post-injection. Flies were weighed per group after the assay; the total weight of the flies was divided by the number of individuals. Assay data were analyzed using Prism v10 (Graphpad). The paralytic dose, PD_50_, was calculated from the righting and climbing assays by fitting a nonlinear regression model with a variable slope (4 parameters). The climbing assay was performed twice and the negative logarithmic values (pPD_50_) averaged across the 2 experiments. The final PD_50_ was calculated by taking the antilog of the average pPD_50_.

### Venom composition annotation, alignments, and phylogenetic reconstructions

To annotate proteins to the family level, we used a combination of approaches. Proteins with signal peptides were identified using both SignalP 5.0 (Almagro Armenteros et al. 2019) and SignalP 6.0 (Teufel et al. 2022). As a base for protein family annotation, we used InterProScan v5.62-94 (Jones et al. 2014; Blum et al. 2025) and supplemented this with searches against UniProtKB (The UniProt 2017) and the FoldSeek server (van Kempen et al. 2024). For proteins and peptides where we wanted to check putative cleavage site analysis, we used ProP v1.0 (Duckert et al. 2004). Protein family names were assigned based on the best hit or when complementary analyses yielded consistent results. Proteins labeled as “uncharacterized” received this designation because they produced no significant hits.

To assign venom-identified components to groups within the venom dataset and to support family reconstructions across the complete annotation, we performed clustering analysis using MMseqs2 v17-b804f (Steinegger and Söding 2017) (search mode with sensitivity 9 and 2 iterations). The resulting clusters were manually curated to refine family assignments. For phylogenetic analysis, protein families were aligned by extracting domains of interest and aligning them with MAFFT v7.490 (Katoh and Standley 2013) using the L-INS-i algorithm. Maximum likelihood phylogenetic reconstructions were performed using IQ-TREE v2.2.0 (Minh et al. 2013) with 1,000 UFBoot replicates (Hoang et al. 2018) and ModelFinder Plus (Kalyaanamoorthy et al. 2017) for model selection. Phylogenetic trees were visualized using phytools v2.0 (Revell 2024) and protein structures were predicted using ColabFold v1.5.2 (Jumper et al. 2021; Mirdita et al. 2022; Kim et al. 2025).

### Venom composition comparison to previously reported Neuroptera venom components

To compare our venom annotation to that previously reported by Fischer et al. (2024), we analyzed their reported *Euroleon nostras* annotation (Supplementary Data S1 in Fischer et al. 2024) and *C. carnea* annotation (Supplementary Data S2 in Fischer et al. 2024). Transcripts were selected based on proteomic support and the presence of a predicted signal peptide, including those with uncertain predictions marked with a question mark reported in Fischer et al. (2024). We also included the hemolysin transcripts that were identified. The transcripts were mapped to their appropriate reference genomes, with *C. carnea* transcripts mapped to the *C. carnea* reported here (BM3) and *E. nostras* transcripts mapped to the available *E. nostras* genome (Fischer et al. 2024) using miniprot v0.15-r270 (Li 2023 ). The resulting annotations were processed using AGAT in 4 sequential steps. First, agat_sp_fix_overlaping_genes.pl was used to identify and merge gene features with overlapping CDS regions. Second, agat_sp_keep_longest_isoform.pl was applied to retain only the longest isoform per locus. Third, agat_sp_fix_cds_phases.pl was used to fix any erroneous CDS phases. Finally, agat_sp_extract_sequences.pl was used to extract the processed sequences. For comparison between the 2 *C. carnea* datasets, gene presence was assessed manually using the Integrative Genomics Viewer (Robinson et al. 2011). For orthogroup comparisons between the *C. carnea* and *E. nostras* datasets, orthogroups were assigned using OrthoFinder v2.5.5 (Emms and Kelly 2019).

### Differential gene expression and tissue clustering

For differential gene expression analysis, read counts were generated using featureCounts v2.0.4 (Liao et al. 2014). Gene-level read counts were obtained by counting reads mapping to CDS features (“-t CDS”) using the gene ID attribute (“-g gene_id”) from our *C. carnea* annotation. FeatureCounts was configured for paired-end libraries (“-p”) with fragment counting (“–countReadPairs”). Due to one CVG sample failing sequencing, only 2 CVG replicates were available. The resulting count matrix was processed using the edgeR package v4.2.1 (Chen et al. 2025) in R. Library sizes were normalized using TMM normalization. Data quality and sample relationships were assessed using multidimensional scaling (MDS) plots from edgeR and hierarchical clustering analysis using Ward.D2 linkage on Euclidean distances of log2 CPM values between samples. Sample similarity was visualized using Pearson correlation heatmaps of log2 CPM values with Ward.D2 clustering using the ComplexHeatmap package (Gu et al. 2016).

Samples were grouped into venom-producing tissues (MXVG, CVG) versus nonvenom tissues (CNS, DG, MG). Differential gene expression was tested using edgeR quasi-likelihood pipeline (glmQLFit/glmTreat) with a log2 fold change threshold of log2(1.2) and FDR < 0.05. Expression patterns of significantly differentially expressed genes with signal peptides were visualized using hierarchical clustering heatmaps using the pheatmap package v1.0.12 and the Ward.D2 clustering method.

### Alternative splicing analysis

To quantify the number of alternative splicing events, we used the generateEvents function of SUPPA2 (Trincado et al. 2018) using our *C. carnea* annotation. In short, SUPPA2 generateEvents uses exon-level information from the transcript and gene information and classifies events into skipping exon (SE), alternative 5′/3′ splice sites (A5/A3), mutually exclusive exons (MX), retained intron (RI), and alternative first/last exons (AF/AL). We then categorized splicing events by the number of amino acid (AA) changes introduced. Isoforms of genes with splicing events identified by SUPPA2 were extracted and aligned per gene using MAFFT v7.490 using the –auto mode. We then counted changes introduced in the number of amino acids and indels, separating the number of events that included all changes and the number of events that resulted in indels > 3 amino acids.

To examine the significance of splicing events and if we observe any isoform switching between our tissues, we first performed differential transcript expression using Salmon v1.10.3 (Patro et al. 2017) to generate TPM values for our tissue samples. We then performed isoform switching analysis using IsoformSwitchAnalyzeR (Vitting-Seerup and Sandelin 2019), applying the default preFilter command, followed by satuRn (Gilis et al. 2021) to test for significant isoform switching of our venom proteome-identified genes between venom-producing and nonvenom tissues.

To explore convergent recruitment of the venom Serpin gene across venomous insect lineages, we collated isoforms of this gene from several venomous and 2 nonvenomous insect lineages (SI Table S5). We then aligned the Serpin domains using MAFFT v7.490 with the L-INS-i algorithm. Maximum likelihood phylogenetic reconstructions were performed using IQ-TREE v2.2.0 with 1,000 UFBoot replicates and ModelFinder Plus for model selection.

## Supplementary Material

msaf326_Supplementary_Data

## Data Availability

The draft assembly for BM3 is available at NCBI (PRJNA731104). Raw Iso-Seq reads and, the whole body and tissue RNA-Seq data is available at NCBI (PRJNA731104). SI Data S1 and S2 are available as supplementary files. The *C. carnea* genome annotation, draft assembly, and SI Video S1 and S2 are available at Figshare (https://doi.org/10.6084/m9.figshare.c.7998802). The mass spectrometry proteomics has been deposited to the ProteomeXchange Consortium via the PRIDE partner repository under the project accession PXD070278.

## Supplementary material

SI Figures and Tables. Document containing Figures S1–10 and Tables S1–5.

SI Video S1. Venom extraction from *C. carnea*.

SI Video S2. Predation on *D. melanogaster* larva by first-instar *C. carnea* larva.

SI Data S1. Text file with *C. carnea* venom annotation and proteomic summary.

SI Data S2. Excel file with genome alignment of previously published *C. carnea* venom genes to genes reported here.

SI Data S3. CLIP and SP phylogeny treefile in newick format.

SI Data S4. Serpin phylogeny treefile in newick format.

## References

[msaf326-B1] Almagro Armenteros JJ et al Signalp 5.0 improves signal peptide predictions using deep neural networks. Nat Biotechnol. 2019:37:420–423. 10.1038/s41587-019-0036-z. 30778233

[msaf326-B2] Almeida DD et al Tracking the recruitment and evolution of snake toxins using the evolutionary context provided by the *Bothrops jararaca* genome. Proc Natl Acad Sci U S A. 2021:118:e2015159118. 10.1073/pnas.2015159118. 33972420 PMC8157943

[msaf326-B3] Barua A, Mikheyev AS. An ancient, conserved gene regulatory network led to the rise of oral venom systems. Proc Natl Acad Sci U S A. 2021:118:e2021311118. 10.1073/pnas.2021311118. 33782124 PMC8040605

[msaf326-B4] Beutel RG, Friedrich F, Aspöck U. The larval head of Nevrorthidae and the phylogeny of Neuroptera (Insecta). Zool J Linn Soc. 2010:158:533–562. 10.1111/j.1096-3642.2009.00560.x.

[msaf326-B5] Blank S et al Vitellogenins are new high molecular weight components and allergens (Api m 12 and Ves v 6) of *Apis mellifera* and *Vespula vulgaris* venom. PLoS One. 2013:8:e62009. 10.1371/journal.pone.0062009. 23626765 PMC3633918

[msaf326-B6] Bleau JR, Gaur N, Fu Y, Bos JIB. Unveiling the slippery secrets of saliva: effector proteins of phloem-feeding insects. Mol Plant Microbe Interact. 2024:37:211–219. 10.1094/MPMI-10-23-0167-FI. 38148271

[msaf326-B7] Blum M et al InterPro: the protein sequence classification resource in 2025. Nucleic Acids Res. 2025:53:D444–D456. 10.1093/nar/gkae1082. 39565202 PMC11701551

[msaf326-B8] Buonocore F et al Attacins: a promising class of insect antimicrobial peptides. Antibiotics (Basel). 2021:10:212. 10.3390/antibiotics10020212. 33672685 PMC7924397

[msaf326-B9] Candiano G et al Blue silver: a very sensitive colloidal Coomassie G-250 staining for proteome analysis. Electrophoresis. 2004:25:1327–1333. 10.1002/elps.200305844. 15174055

[msaf326-B10] Casewell NR, Wüster W, Vonk FJ, Harrison RA, Fry BG. Complex cocktails: the evolutionary novelty of venoms. Trends Ecol Evol. 2013:28:219–229. 10.1016/j.tree.2012.10.020. 23219381

[msaf326-B11] Challis R, Richards E, Rajan J, Cochrane G, Blaxter M. BlobToolKit—interactive quality assessment of genome assemblies. G3 (Bethesda). 2020:10:1361–1374. 10.1534/g3.119.400908. 32071071 PMC7144090

[msaf326-B12] Chen J et al SjAPI-2 is the first member of a new neurotoxin family with Ascaris-type fold and KCNQ1 inhibitory activity. Int J Biol Macromol. 2015:79:504–510. 10.1016/j.ijbiomac.2015.05.027. 26014142

[msaf326-B13] Chen Y, Chen L, Lun ATL, Baldoni PL, Smyth GK. Edger v4: powerful differential analysis of sequencing data with expanded functionality and improved support for small counts and larger datasets. Nucleic Acids Res. 2025:53:gkaf018. 10.1093/nar/gkaf018. 39844453 PMC11754124

[msaf326-B14] Chen Z et al SjAPI, the first functionally characterized ascaris-type protease inhibitor from animal venoms. PLoS One. 2013:8:e57529. 10.1371/journal.pone.0057529. 23533574 PMC3606364

[msaf326-B15] Cheng H, Concepcion GT, Feng X, Zhang H, Li H. Haplotype-resolved de novo assembly using phased assembly graphs with hifiasm. Nat Methods. 2021:18:170–175. 10.1038/s41592-020-01056-5. 33526886 PMC7961889

[msaf326-B16] Cox J, Mann M. MaxQuant enables high peptide identification rates, individualized p.p.b.-range mass accuracies and proteome-wide protein quantification. Nat Biotechnol. 2008:26:1367–1372. 10.1038/nbt.1511. 19029910

[msaf326-B17] Crowley LM . The genome sequence of the common green lacewing, *Chrysoperla carnea* (Stephens, 1836). Wellcome Open Res. 2021:6:334. 10.12688/wellcomeopenres.17455.1. 37089663 PMC10116181

[msaf326-B18] Dainat J et al Zenodo. 2024. 10.5281/zenodo.13799920.

[msaf326-B19] Danecek P et al Twelve years of SAMtools and BCFtools. GigaScience. 2021:10:giab008. 10.1093/gigascience/giab008. 33590861 PMC7931819

[msaf326-B20] Dash TS et al A centipede toxin family defines an ancient class of CSαβ defensins. Structure. 2019:27:315–326.e7. 10.1016/j.str.2018.10.022. 30554841

[msaf326-B21] Dashevsky D et al Functional and proteomic insights into Aculeata venoms. Toxins (Basel). 2023:15:224. 10.3390/toxins15030224. 36977115 PMC10053895

[msaf326-B22] Drukewitz SH et al A dipteran's novel sucker punch: evolution of arthropod atypical venom with a neurotoxic component in robber flies (Asilidae, Diptera). Toxins (Basel). 2018:10:29. 10.3390/toxins10010029. 29303983 PMC5793116

[msaf326-B23] Duckert P, Brunak S, Blom N. Prediction of proprotein convertase cleavage sites. Protein Eng Des Sel. 2004:17:107–112. 10.1093/protein/gzh013. 14985543

[msaf326-B24] Emms DM, Kelly S. OrthoFinder: phylogenetic orthology inference for comparative genomics. Genome Biol. 2019:20:238. 10.1186/s13059-019-1832-y. 31727128 PMC6857279

[msaf326-B25] Fischer ML et al Divergent venom effectors correlate with ecological niche in neuropteran predators. Commun Biol. 2024:7:981. 10.1038/s42003-024-06666-9. 39134630 PMC11319779

[msaf326-B26] Fry B et al The toxicogenomic multiverse: convergent recruitment of proteins into animal venoms. Annu Rev Genomics Hum Genet. 2009:10:483–511. 10.1146/annurev.genom.9.081307.164356. 19640225

[msaf326-B27] Gabriel L et al BRAKER3: fully automated genome annotation using RNA-seq and protein evidence with GeneMark-ETP, AUGUSTUS, and TSEBRA. Genome Res. 2024:34:769–777. 10.1101/gr.278090.123. 38866550 PMC11216308

[msaf326-B28] Gilis J, Vitting-Seerup K, Van den Berge K, Clement L. Saturn: scalable analysis of differential transcript usage for bulk and single-cell RNA-sequencing applications. F1000Res. 2021:10:374. 10.12688/f1000research.51749.1. 36762203 PMC9892655

[msaf326-B29] Gong H et al Characterization of a single-domain von Willebrand factor type C protein (HaSVC) from the salivary gland of the tick *Hyalomma asiaticum*. Exp Parasitol. 2022:232:108190. 10.1016/j.exppara.2021.108190. 34848245

[msaf326-B30] Gopalan SS et al Origins, genomic structure and copy number variation of snake venom myotoxins. Toxicon. 2022:216:92–106. 10.1016/j.toxicon.2022.06.014. 35820472

[msaf326-B31] Grabherr MG et al Full-length transcriptome assembly from RNA-Seq data without a reference genome. Nat Biotechnol. 2011:29:644–652. 10.1038/nbt.1883. 21572440 PMC3571712

[msaf326-B32] Gu Z, Eils R, Schlesner M. Complex heatmaps reveal patterns and correlations in multidimensional genomic data. Bioinformatics. 2016:32:2847–2849. 10.1093/bioinformatics/btw313. 27207943

[msaf326-B33] Haas BJ et al Improving the *Arabidopsis* genome annotation using maximal transcript alignment assemblies. Nucleic Acids Res. 2003:31:5654–5666. 10.1093/nar/gkg770. 14500829 PMC206470

[msaf326-B34] Haas BJ et al De novo transcript sequence reconstruction from RNA-seq using the Trinity platform for reference generation and analysis. Nat Protoc. 2013:8:1494–1512. 10.1038/nprot.2013.084. 23845962 PMC3875132

[msaf326-B35] Hekmat-Scafe DS, Scafe CR, McKinney AJ, Tanouye MA. Genome-wide analysis of the odorant-binding protein gene family in *Drosophila melanogaster*. Genome Res. 2002:12:1357–1369. 10.1101/gr.239402. 12213773 PMC186648

[msaf326-B36] Hoang DT, Chernomor O, von Haeseler A, Minh BQ, Vinh LS. UFBoot2: improving the ultrafast bootstrap approximation. Mol Biol Evol. 2018:35:518–522. 10.1093/molbev/msx281. 29077904 PMC5850222

[msaf326-B37] Holehouse AS, Kragelund BB. The molecular basis for cellular function of intrinsically disordered protein regions. Nat Rev Mol Cell Biol. 2024:25:187–211. 10.1038/s41580-023-00673-0. 37957331 PMC11459374

[msaf326-B38] Jenner RA, Casewell NR, Undheim EAB. What is animal venom? Rethinking a manipulative weapon. Trends Ecol Evol. 2025:40:852–861. 10.1016/j.tree.2025.05.009. 40544034

[msaf326-B39] Jones P et al InterProScan 5: genome-scale protein function classification. Bioinformatics. 2014:30:1236–1240. 10.1093/bioinformatics/btu031. 24451626 PMC3998142

[msaf326-B40] Jumper J et al Highly accurate protein structure prediction with AlphaFold. Nature. 2021:596:583–589. 10.1038/s41586-021-03819-2. 34265844 PMC8371605

[msaf326-B41] Kalyaanamoorthy S, Minh BQ, Wong TKF, von Haeseler A, Jermiin LS. ModelFinder: fast model selection for accurate phylogenetic estimates. Nat Methods. 2017:14:587–589. 10.1038/nmeth.4285. 28481363 PMC5453245

[msaf326-B42] Kanost MR, Jiang H. Clip-domain serine proteases as immune factors in insect hemolymph. Curr Opin Insect Sci. 2015:11:47–55. 10.1016/j.cois.2015.09.003. 26688791 PMC4680995

[msaf326-B43] Katoh K, Standley DM. MAFFT multiple sequence alignment software version 7: improvements in performance and usability. Mol Biol Evol. 2013:30:772–780. 10.1093/molbev/mst010. 23329690 PMC3603318

[msaf326-B44] Kern O et al The structures of two salivary proteins from the west Nile vector *Culex quinquefasciatus* reveal a beta-trefoil fold with putative sugar binding properties. Curr Res Struct Biol. 2021:3:95–105. 10.1016/j.crstbi.2021.03.001. 34235489 PMC8244437

[msaf326-B45] Kim D, Paggi JM, Park C, Bennett C, Salzberg SL. Graph-based genome alignment and genotyping with HISAT2 and HISAT-genotype. Nat Biotechnol. 2019:37:907–915. 10.1038/s41587-019-0201-4. 31375807 PMC7605509

[msaf326-B46] Kim G et al Easy and accurate protein structure prediction using ColabFold. Nat Protoc. 2025:20:620–642. 10.1038/s41596-024-01060-5. 39402428

[msaf326-B47] King JG, Vernick KD, Hillyer JF. Members of the salivary gland surface protein (SGS) family are major immunogenic components of mosquito saliva. J Biol Chem. 2011:286:40824–40834. 10.1074/jbc.M111.280552. 21965675 PMC3220476

[msaf326-B48] Koludarov I et al Domain loss enabled evolution of novel functions in the snake three-finger toxin gene superfamily. Nat Commun. 2023:14:4861. 10.1038/s41467-023-40550-0. 37567881 PMC10421932

[msaf326-B49] Krämer J, Hölker P, Predel R. How to overcome a snail? Identification of putative neurotoxins of snail-feeding firefly larvae (Coleoptera: Lampyridae, *Lampyris noctiluca*). Toxins (Basel). 2024:16:272. 10.3390/toxins16060272. 38922166 PMC11209139

[msaf326-B50] Krueger F et al Zenodo. 2023. 10.5281/zenodo.7598955.

[msaf326-B51] Kuznetsov D et al OrthoDB v11: annotation of orthologs in the widest sampling of organismal diversity. Nucleic Acids Res. 2023:51:D445–D451. 10.1093/nar/gkac998. 36350662 PMC9825584

[msaf326-B52] Li D et al Unearthing underground predators: the head morphology of larvae of the moth lacewing genus *Ithone* Newman (Neuroptera: Ithonidae) and its functional and phylogenetic implications. Syst Entomol. 2022:47:618–636. 10.1111/syen.12556.

[msaf326-B53] Li H . Protein-to-genome alignment with miniprot. Bioinformatics. 2023:39:btad014. 10.1093/bioinformatics/btad014. 36648328 PMC9869432

[msaf326-B54] Liang W et al A venom serpin from the assassin bug *Sycanus croceovittatus* exhibiting inhibitory effects on melanization, development, and insecticidal activity towards its prey. Pestic Biochem Physiol. 2025:209:106322. 10.1016/j.pestbp.2025.106322. 40082049

[msaf326-B55] Liao Y, Smyth GK, Shi W. featureCounts: an efficient general purpose program for assigning sequence reads to genomic features. Bioinformatics. 2014:30:923–930. 10.1093/bioinformatics/btt656. 24227677

[msaf326-B56] Liu S, Xia X, Calvo E, Zhou ZH. Native structure of mosquito salivary protein uncovers domains relevant to pathogen transmission. Nat Commun. 2023:14:899. 10.1038/s41467-023-36577-y. 36797290 PMC9935623

[msaf326-B57] Lomonte B, Rangel J. Snake venom Lys49 myotoxins: from phospholipases A2 to non-enzymatic membrane disruptors. Toxicon. 2012:60:520–530. 10.1016/j.toxicon.2012.02.007. 22781132

[msaf326-B58] Manni M, Berkeley MR, Seppey M, Simão FA, Zdobnov EM. BUSCO update: novel and streamlined workflows along with broader and deeper phylogenetic coverage for scoring of eukaryotic, prokaryotic, and viral genomes. Mol Biol Evol. 2021:38:4647–4654. 10.1093/molbev/msab199. 34320186 PMC8476166

[msaf326-B59] Martin M . Cutadapt removes adapter sequences from high-throughput sequencing reads. EMBnet J. 2011:17:10–12. 10.14806/ej.17.1.200.

[msaf326-B60] Martinson EO, Mrinalini M, Kelkar YD, Chang C-H, Werren JH. The evolution of venom by co-option of single-copy genes. Curr Biol. 2017:27:2007–2013.e8. 10.1016/j.cub.2017.05.032. 28648823 PMC5719492

[msaf326-B61] Matsuda K et al Purification and characterization of a paralytic polypeptide from larvae of Myrmeleon bore. Biochem Biophys Res Commun. 1995:215:167–171. 10.1006/bbrc.1995.2448. 7575586

[msaf326-B62] Michel Y et al The putative serine protease inhibitor Api m 6 from Apis mellifera venom: recombinant and structural evaluation. J Investig Allergol Clin Immunol. 2012:22:476–484. 23397669

[msaf326-B63] Minh BQ, Nguyen MAT, von Haeseler A. Ultrafast approximation for phylogenetic bootstrap. Mol Biol Evol. 2013:30:1188–1195. 10.1093/molbev/mst024. 23418397 PMC3670741

[msaf326-B64] Mirdita M et al ColabFold: making protein folding accessible to all. Nat Methods. 2022:19:679–682. 10.1038/s41592-022-01488-1. 35637307 PMC9184281

[msaf326-B65] Moneo I, Vega JM, Caballero ML, Vega J, Alday E. Isolation and characterization of Tha p 1, a major allergen from the pine processionary caterpillar *Thaumetopoea pityocampa*. Allergy. 2003:58:34–37. 10.1034/j.1398-9995.2003.23724.x. 12580804

[msaf326-B66] Moran Y, Fredman D, Szczesny P, Grynberg M, Technau U. Recurrent horizontal transfer of bacterial toxin genes to eukaryotes. Mol Biol Evol. 2012:29:2223–2230. 10.1093/molbev/mss089. 22411854 PMC3424411

[msaf326-B67] Mourão CB, Schwartz EF. Protease inhibitors from marine venomous animals and their counterparts in terrestrial venomous animals. Mar Drugs. 2013:11:2069–2112. 10.3390/md11062069. 23771044 PMC3721222

[msaf326-B68] Nachtigall PG et al The gene regulatory mechanisms shaping the heterogeneity of venom production in the Cape coral snake. Genome Biol. 2025a:26:130. 10.1186/s13059-025-03602-w. 40390047 PMC12087220

[msaf326-B69] Nachtigall PG et al A segregating structural variant defines novel venom phenotypes in the eastern diamondback rattlesnake. Mol Biol Evol. 2025b:42:msaf058. 10.1093/molbev/msaf058. 40101100 PMC11965796

[msaf326-B70] Nachtigall PG, Kashiwabara AY, Durham AM. Codan: predictive models for precise identification of coding regions in eukaryotic transcripts. Brief Bioinform. 2021:22:bbaa045. 10.1093/bib/bbaa045. 32460307 PMC8138839

[msaf326-B71] Nishiwaki H et al Purification and functional characterization of insecticidal sphingomyelinase C produced by *Bacillus cereus*. Eur J Biochem. 2004:271:601–606. 10.1111/j.1432-1033.2003.03962.x. 14728687

[msaf326-B72] Nishiwaki H, Ito K, Shimomura M, Nakashima K, Matsuda K. Insecticidal bacteria isolated from predatory larvae of the antlion species *Myrmeleon bore* (Neuroptera: Myrmeleontidae). J Invertebr Pathol. 2007:96:80–88. 10.1016/j.jip.2007.02.007. 17399737

[msaf326-B73] Ogawa T et al Alternative mRNA splicing in three venom families underlying a possible production of divergent venom proteins of the habu snake, *Protobothrops flavoviridis*. Toxins (Basel). 2019:11:581. 10.3390/toxins11100581. 31600994 PMC6832531

[msaf326-B74] Pan C-T et al The evolution and structure of snake venom phosphodiesterase (svPDE) highlight its importance in venom actions. eLife. 2023:12:e83966. 10.7554/eLife.83966. 37067034 PMC10121219

[msaf326-B75] Patro R, Duggal G, Love MI, Irizarry RA, Kingsford C. Salmon provides fast and bias-aware quantification of transcript expression. Nat Methods. 2017:14:417–419. 10.1038/nmeth.4197. 28263959 PMC5600148

[msaf326-B76] Perez-Riverol A, Lasa AM, Dos Santos-Pinto JRA, Palma MS. Insect venom phospholipases A1 and A2: roles in the envenoming process and allergy. Insect Biochem Mol Biol. 2019:105:10–24. 10.1016/j.ibmb.2018.12.011. 30582958

[msaf326-B77] Pertea M et al StringTie enables improved reconstruction of a transcriptome from RNA-seq reads. Nat Biotechnol. 2015:33:290–295. 10.1038/nbt.3122. 25690850 PMC4643835

[msaf326-B78] Pineda SS et al Structural venomics reveals evolution of a complex venom by duplication and diversification of an ancient peptide-encoding gene. Proc Natl Acad Sci U S A. 2020:117:11399–11408. 10.1073/pnas.1914536117. 32398368 PMC7260951

[msaf326-B79] Qian C et al Identification of a small pacifastin protease inhibitor from *Nasonia vitripennis* venom that inhibits humoral immunity of host (*Musca domestica*). Toxicon. 2017:131:54–62. 10.1016/j.toxicon.2017.03.005. 28283430

[msaf326-B80] Quinlan AR, Hall IM. BEDTools: a flexible suite of utilities for comparing genomic features. Bioinformatics. 2010:26:841–842. 10.1093/bioinformatics/btq033. 20110278 PMC2832824

[msaf326-B81] Reichhardt MP et al An inhibitor of complement C5 provides structural insights into activation. Proc Natl Acad Sci U S A. 2020:117:362–370. 10.1073/pnas.1909973116. 31871188 PMC6955305

[msaf326-B82] Revell LJ . Phytools 2.0: an updated R ecosystem for phylogenetic comparative methods (and other things). PeerJ. 2024:12:e16505. 10.7717/peerj.16505. 38192598 PMC10773453

[msaf326-B83] Rihani K, Ferveur JF, Briand L. The 40-year mystery of insect odorant-binding proteins. Biomolecules. 2021:11:509. 10.3390/biom11040509. 33808208 PMC8067015

[msaf326-B84] Robinson JT et al Integrative genomics viewer. Nat Biotechnol. 2011:29:24–26. 10.1038/nbt.1754. 21221095 PMC3346182

[msaf326-B85] Robinson SD et al A comprehensive portrait of the venom of the giant red bull ant *Myrmecia gulosa* reveals a hyperdiverse hymenopteran toxin gene family. Sci Adv. 2018:4:eaau4640. 10.1126/sciadv.aau4640. 30214940 PMC6135544

[msaf326-B86] Robinson SD et al Intra-colony venom diversity contributes to maintaining eusociality in a cooperatively breeding ant. BMC Biol. 2023:21:5. 10.1186/s12915-022-01507-9. 36617555 PMC9827630

[msaf326-B87] Romeis B . Mikroskopische technik. Oldenbourg Verlag; 1989.

[msaf326-B88] Ruder T et al Molecular phylogeny and evolution of the proteins encoded by coleoid (cuttlefish, octopus, and squid) posterior venom glands. J Mol Evol. 2013:76:192–204. 10.1007/s00239-013-9552-5. 23456102

[msaf326-B89] Schendel V et al Exaptation of an evolutionary constraint enables behavioural control over the composition of secreted venom in a giant centipede. Nat Ecol Evol. 2025:9:73–86. 10.1038/s41559-024-02556-9. 39496866

[msaf326-B90] Schendel V, Rash LD, Jenner RA, Undheim EAB. The diversity of venom: the importance of behavior and venom system morphology in understanding its ecology and evolution. Toxins (Basel). 2019:11:666. 10.3390/toxins11110666. 31739590 PMC6891279

[msaf326-B91] Schmidt JO . The sting of the wild. Johns Hopkins University Press; 2016.

[msaf326-B92] Seldeslachts A et al Exploring oak processionary caterpillar induced lepidopterism (part 1): unveiling molecular insights through transcriptomics and proteomics. Cell Mol Life Sci. 2024:81:311. 10.1007/s00018-024-05330-z. 39066932 PMC11335235

[msaf326-B93] Sombke A, Lipke E, Michalik P, Uhl G, Harzsch S. Potential and limitations of X-ray micro-computed tomography in arthropod neuroanatomy: a methodological and comparative survey. J Comp Neurol. 2015:523:1281–1295. 10.1002/cne.23741. 25728683 PMC4409823

[msaf326-B94] Steinegger M, Söding J. MMseqs2 enables sensitive protein sequence searching for the analysis of massive data sets. Nat Biotechnol. 2017:35:1026–1028. 10.1038/nbt.3988. 29035372

[msaf326-B95] Sun JS, Xiao S, Carlson JR. The diverse small proteins called odorant-binding proteins. Open Biol. 2018:8:180208. 10.1098/rsob.180208. 30977439 PMC6303780

[msaf326-B96] Teufel F et al Signalp 6.0 predicts all five types of signal peptides using protein language models. Nat Biotechnol. 2022:40:1023–1025. 10.1038/s41587-021-01156-3. 34980915 PMC9287161

[msaf326-B97] The UniProt C . UniProt: the universal protein knowledgebase. Nucleic Acids Res. 2017:45:D158–D169. 10.1093/nar/gkw1099. 27899622 PMC5210571

[msaf326-B98] Touchard A et al Elucidation of the unexplored biodiversity of ant venom peptidomes via MALDI–TOF mass spectrometry and its application for chemotaxonomy. J Proteomics. 2014:105:217–231. 10.1016/j.jprot.2014.01.009. 24456813

[msaf326-B99] Touchard A et al The genome of the ant *Tetramorium bicarinatum* reveals a tandem organization of venom peptides genes allowing the prediction of their regulatory and evolutionary profiles. BMC Genomics. 2024:25:84. 10.1186/s12864-024-10012-y. 38245722 PMC10800049

[msaf326-B100] Trincado JL et al SUPPA2: fast, accurate, and uncertainty-aware differential splicing analysis across multiple conditions. Genome Biol. 2018:19:40. 10.1186/s13059-018-1417-1. 29571299 PMC5866513

[msaf326-B101] Undheim EAB et al Clawing through evolution: toxin diversification and convergence in the ancient lineage Chilopoda (centipedes). Mol Biol Evol. 2014:31:2124–2148. 10.1093/molbev/msu162. 24847043 PMC4104317

[msaf326-B102] Undheim EAB et al Weaponization of a hormone: convergent recruitment of hyperglycemic hormone into the venom of arthropod predators. Structure. 2015:23:1283–1292. 10.1016/j.str.2015.05.003. 26073605

[msaf326-B103] Undheim EAB, Jenner RA. Phylogenetic analyses suggest centipede venom arsenals were repeatedly stocked by horizontal gene transfer. Nat Commun. 2021:12:818. 10.1038/s41467-021-21093-8. 33547293 PMC7864903

[msaf326-B104] Undheim EAB, Mobli M, King GF. Toxin structures as evolutionary tools: using conserved 3D folds to study the evolution of rapidly evolving peptides. BioEssays. 2016:38:539–548. 10.1002/bies.201500165. 27166747

[msaf326-B105] van Kempen M et al Fast and accurate protein structure search with Foldseek. Nat Biotechnol. 2024:42:243–246. 10.1038/s41587-023-01773-0. 37156916 PMC10869269

[msaf326-B106] Vasilikopoulos A et al An integrative phylogenomic approach to elucidate the evolutionary history and divergence times of Neuropterida (Insecta: Holometabola). BMC Evol Biol. 2020:20:64. 10.1186/s12862-020-01631-6. 32493355 PMC7268685

[msaf326-B107] Vitting-Seerup K, Sandelin A. IsoformSwitchAnalyzeR: analysis of changes in genome-wide patterns of alternative splicing and its functional consequences. Bioinformatics. 2019:35:4469–4471. 10.1093/bioinformatics/btz247. 30989184

[msaf326-B108] Walker AA et al Melt with this kiss: paralyzing and liquefying venom of the assassin bug *Pristhesancus plagipennis* (Hemiptera: Reduviidae). Mol Cell Proteomics. 2017:16:552–566. 10.1074/mcp.M116.063321. 28130397 PMC5383778

[msaf326-B109] Walker AA et al Buzz kill: function and proteomic composition of venom from the giant assassin fly *Dolopus genitalis* (Diptera: Asilidae). Toxins (Basel). 2018a:10:456. 10.3390/toxins10110456. 30400621 PMC6266666

[msaf326-B110] Walker AA et al Entomo-venomics: the evolution, biology and biochemistry of insect venoms. Toxicon. 2018b:154:15–27. 10.1016/j.toxicon.2018.09.004. 30267720

[msaf326-B111] Walker AA et al Missiles of mass disruption: composition and glandular origin of venom used as a projectile defensive weapon by the assassin bug *Platymeris rhadamanthus*. Toxins (Basel). 2019:11:673. 10.3390/toxins11110673. 31752210 PMC6891600

[msaf326-B112] Walker AA et al Horizontal gene transfer underlies the painful stings of asp caterpillars (Lepidoptera: Megalopygidae). Proc Natl Acad Sci U S A. 2023:120:e2305871120. 10.1073/pnas.2305871120. 37428925 PMC10629529

[msaf326-B113] Wang L, Zhu J-Y, Qian C, Fang Q, Ye G-Y. Venom of the parasitoid wasp *Pteromalus puparum* contains an odorant binding protein. Arch Insect Biochem Physiol. 2015:88:101–110. 10.1002/arch.21206. 25256903

[msaf326-B114] Wang Y et al The first chromosome-level genome assembly of a green lacewing *Chrysopa pallens* and its implication for biological control. Mol Ecol Resour. 2022:22:755–767. 10.1111/1755-0998.13503. 34549894 PMC9292380

[msaf326-B115] Yan Z et al A venom serpin splicing isoform of the endoparasitoid wasp *Pteromalus puparum* suppresses host prophenoloxidase cascade by forming complexes with host hemolymph proteinases. J Biol Chem. 2017:292:1038–1051. 10.1074/jbc.M116.739565. 27913622 PMC5247638

[msaf326-B116] Yan Z et al A serpin gene from a parasitoid wasp disrupts host immunity and exhibits adaptive alternative splicing. PLoS Pathog. 2023:19:e1011649. 10.1371/journal.ppat.1011649. 37695779 PMC10513286

[msaf326-B117] Yang L, Qiu L-M, Fang Q, Ye G-Y. A venom protein, Kazal-type serine protease inhibitor, of ectoparasitoid *Pachycrepoideus vindemiae* inhibits the hemolymph melanization of host *Drosophila melanogaster*. Arch Insect Biochem Physiol. 2020:105:e21736. 10.1002/arch.21736. 32918775

[msaf326-B118] Ye X et al Comprehensive isoform-level analysis reveals the contribution of alternative isoforms to venom evolution and repertoire diversity. Genome Res. 2023:33:1554–1567. 10.1101/gr.277707.123. 37798117 PMC10620052

[msaf326-B119] Yoshida N et al Detection of ALMB-toxin in the larval body of Myrmeleon bore by anti-N-terminus peptide antibodies. Biosci Biotechnol Biochem. 1999:63:232–234. 10.1271/bbb.63.232. 10052150

[msaf326-B120] Yoshida N et al Protein function. Chaperonin turned insect toxin. Nature. 2001:411:44. 10.1038/35075148. 11333970

[msaf326-B121] Zeng X-C, Wang S-X, Li W-X. Identification of BmKAPi, a novel type of scorpion venom peptide with peculiar disulfide bridge pattern from *Buthus martensii* Karsch. Toxicon. 2002:40:1719–1722. 10.1016/S0041-0101(02)00134-4. 12457884

[msaf326-B122] Zhang G, Lu Z-Q, Jiang H, Asgari S. Negative regulation of prophenoloxidase (proPO) activation by a clip-domain serine proteinase homolog (SPH) from endoparasitoid venom. Insect Biochem Mol Biol. 2004:34:477–483. 10.1016/j.ibmb.2004.02.009. 15110869

[msaf326-B123] Zhao C et al A massive expansion of effector genes underlies gall-formation in the wheat pest *Mayetiola destructor*. Curr Biol. 2015:25:613–620. 10.1016/j.cub.2014.12.057. 25660540

[msaf326-B124] Zimmermann D, Randolf S, Aspöck U. From chewing to sucking via phylogeny—from sucking to chewing via ontogeny: mouthparts of neuroptera. In: Krenn HW, editor. Insect mouthparts: form, function, development and performance. Springer International Publishing; 2019. p. 361–385.

