# Supplementary material for: The green lacewing venom system and the complex mechanisms underlying its evolution
Source: Mol Biol Evol. 2025 Dec 11;43(1):msaf326. doi: 10.1093/molbev/msaf326 (PMC12758981; doi:10.1093/molbev/msaf326)
Supplement: msaf326_Supplementary_Data [file msaf326_supplementary_data.zip › Maurstad_et_al_2025_Supplementary_material_revised.pdf]

Supplementary material

Multiple Evolutionary Mechanisms Underlie the Venom Composition of the Green Lacewing

Marius F. Maurstad[1], Iris Bea L. Ramiro[1], Jan Philip Oeyen[1,2], Andy Sombke[3], Sebastian Büsse[4], Pedro G. Nachtigall[1], Kjetill S. Jakobsen[1], Eivind A.B. Undheim[1,*]

1 Centre for Ecological and Evolutionary Synthesis, Department of Biosciences, University of Oslo, Oslo, Norway
2 Division of biotechnology and plant health & viruses, bacteria and nematodes in forestry, agriculture and horticulture, Norwegian Institute of Bioeconomy Research (NIBIO), Oslo, Norway
3 Centre for Anatomy and Cell Biology, Cell and Developmental Biology, Medical University of Vienna, Vienna, Austria
4 Cytology and Evolutionary Biology, Zoological Institute and Museum, University of Greifswald, Greifswald, Germany

*Corresponding author. Email: e.a.b.undheim@ibv.uio.no

Supplementary figures

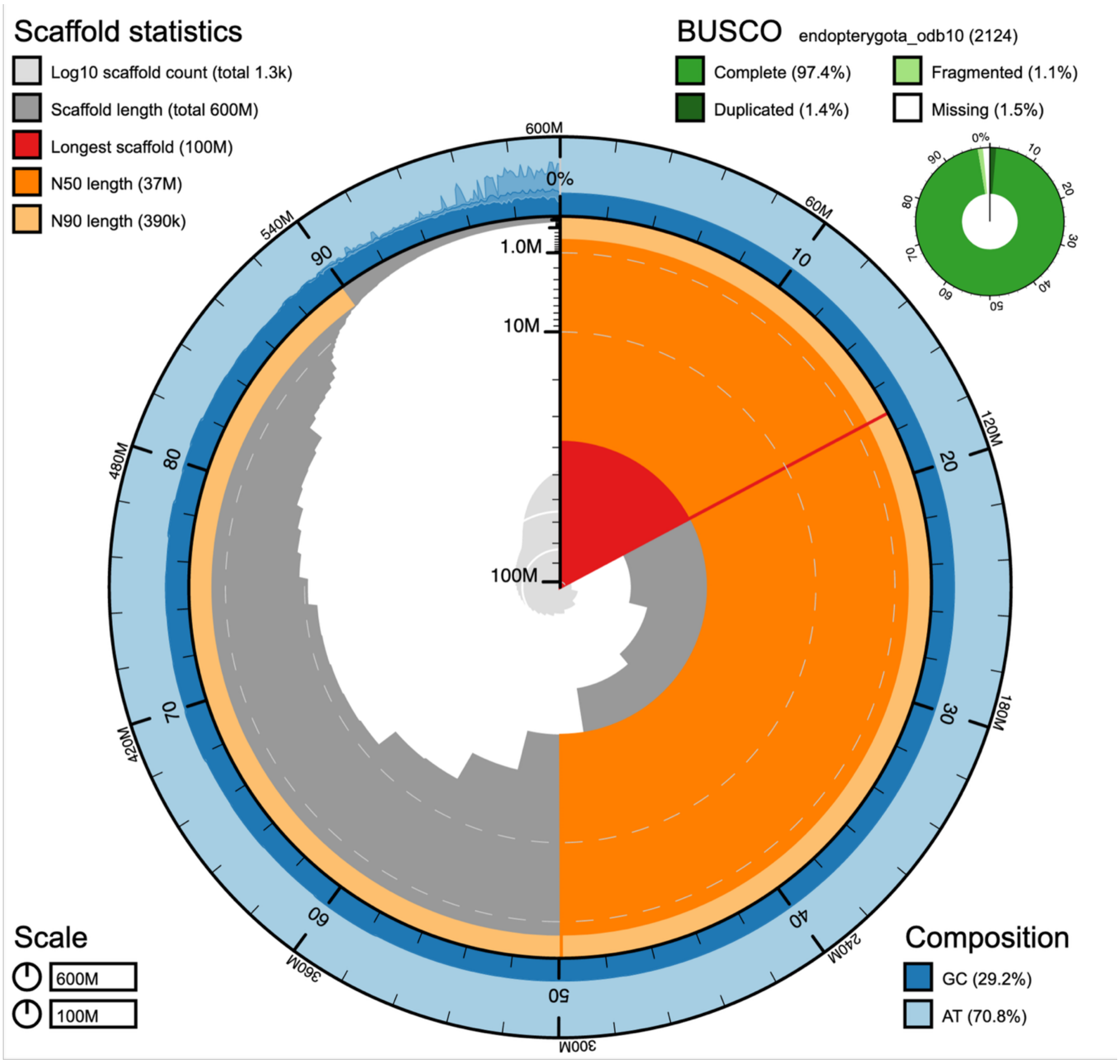

Figure S1. BlobToolKit Snailplot for the male *Chrysoperla carnea* assembly from Oslo (BM3) used in this study. The snailplot show N50 metrics and BUSCO gene completeness.

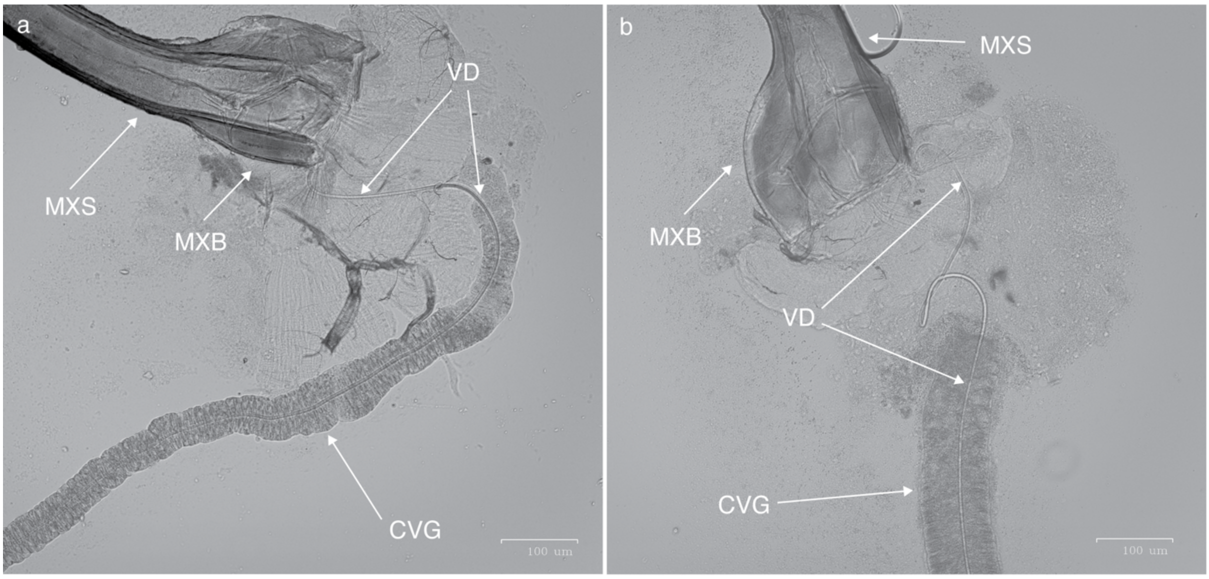

Figure S2. Micro-dissected maxillary stylet and cephalic venom gland. Specimens placed on a slide showing how the lumen connects the cephalic venom gland to the maxillary stylet in **a**) and **b**). Scale bar = 100 µm.

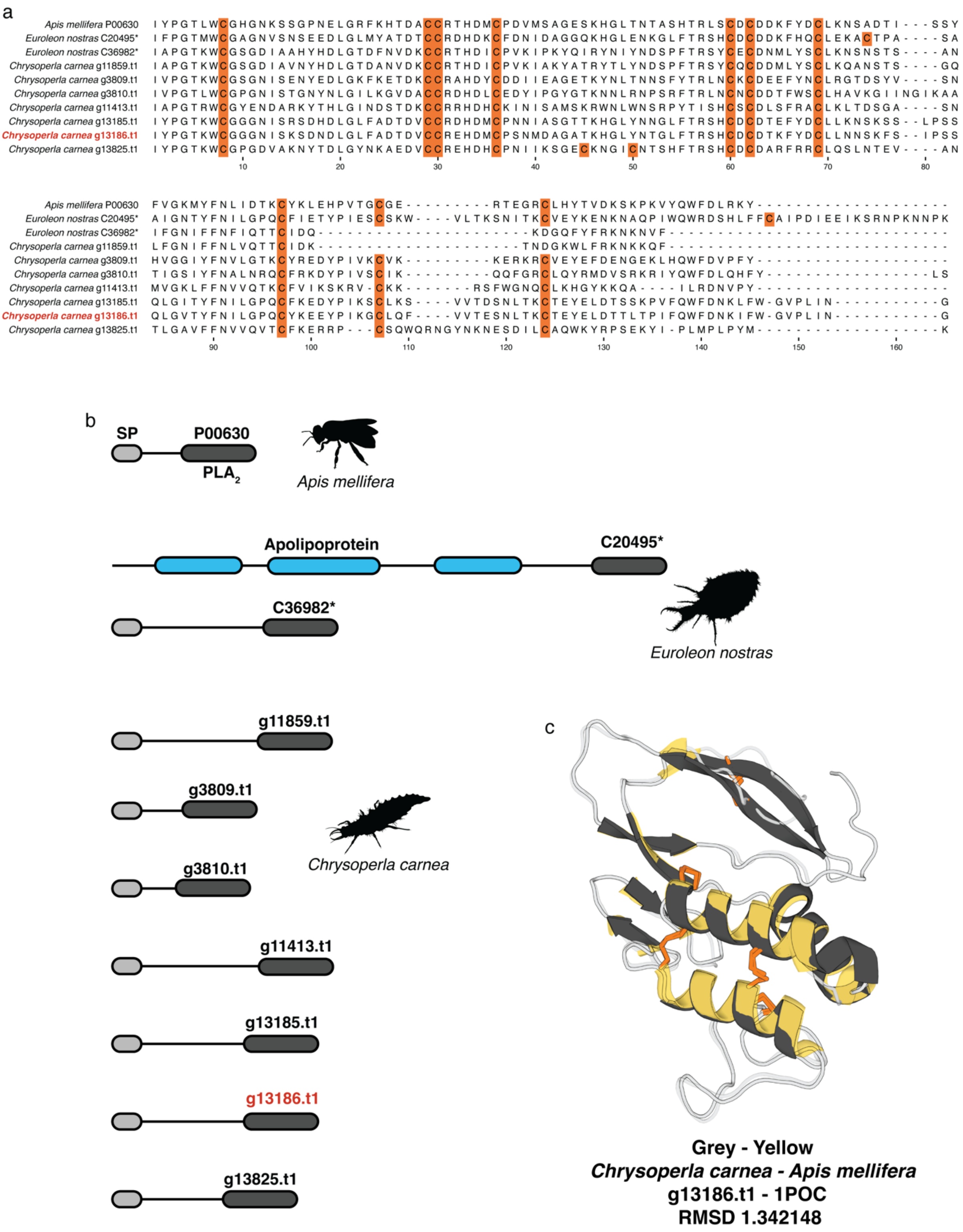

Figure S3. Identification of a PLA2 venom gene in *C. carnea*. a) Amino acid alignment of PLA2 domains identified in the genome annotation of *C. carnea* including the venom proteome identified (red) and the PLA2 identified in *Euroleon nostras* from Fischer et al. 2024 and bee venom PLA2 (UniProt: P00630 - PDB: 1POC). **b**) Protein domain visualization showing signal peptide (light grey box), apolipoprotein domains (blue box) and PLA2 (Dark grey box). **c**) Structural alignment of the *C. carnea* and bee venom PLA2 (1POC). The prediction for the *C. carnea* PLA2 was made with ColabFold.

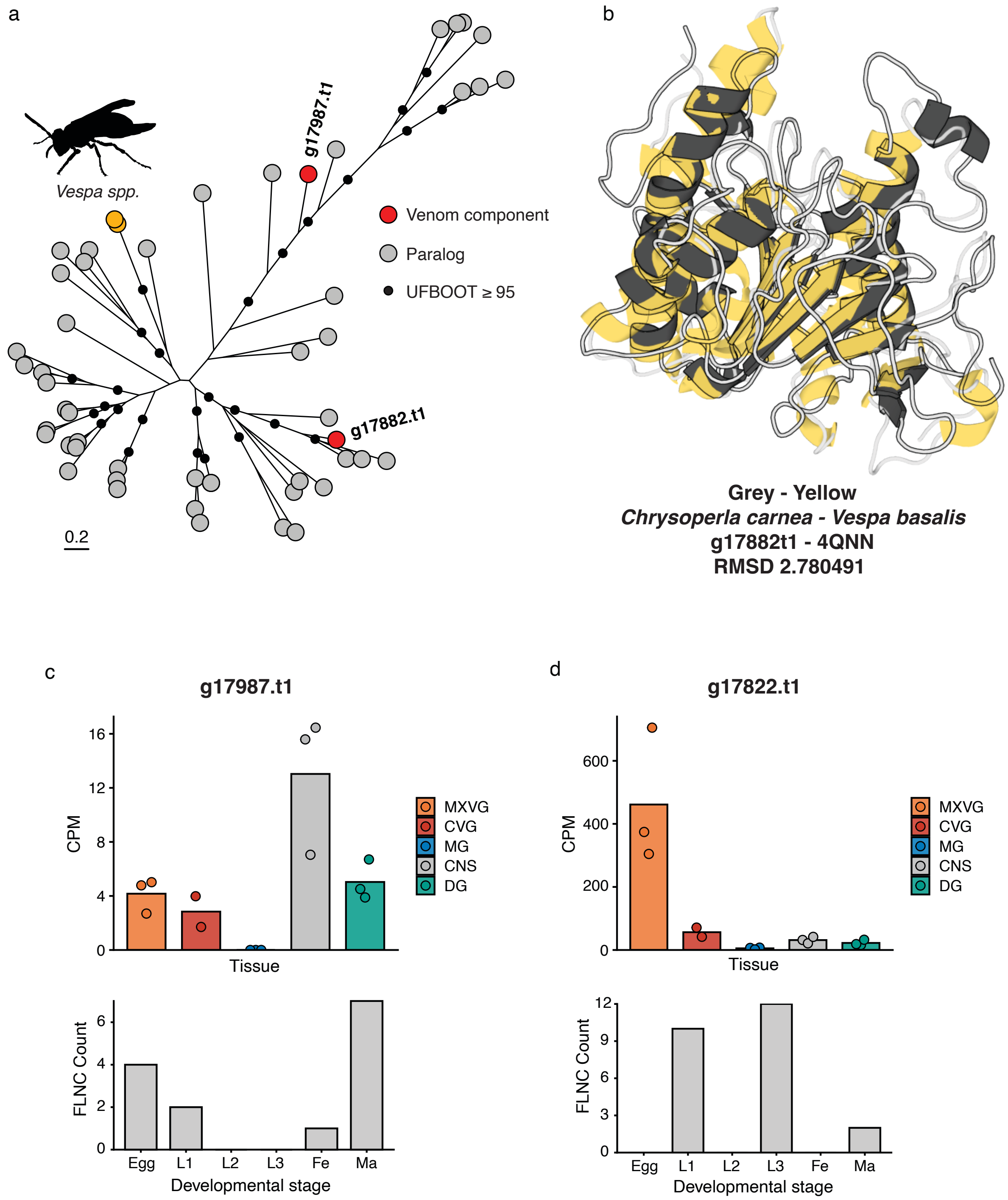

Figure S4. Identification of two PLA1 venom genes in the venom of *C. carnea.* a) Maximum likelihood phylogenetic tree of PLA1 orthologs in the annotation of *C. carnea* and two wasp PLA1 venom components (UniProt: A0A0M3KKW3 and P0DPT0). **b**) Structural alignment of the *C. carnea* and *Vespa basalis* venom PLA1 (4QNN). The prediction for the *C. carnea* PLA1 was made with ColabFold. **c**) and **d**) Tissue expression (top) in CPM (counts per million), and expression across life stages (bottom) shown by FLNC (full-length non-chimeric reads) for the two PLA1 genes identified in the venom proteome. The gene g17987.t1 was only identified in the SDS-PAGE proteomic data.

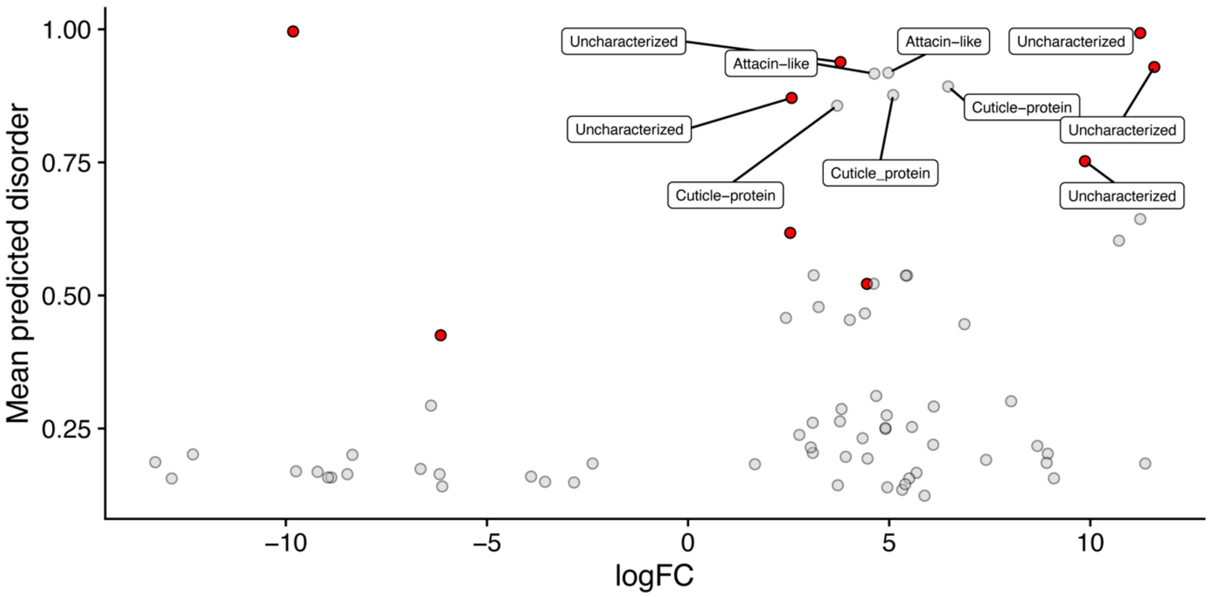

Figure S5. Mean predicted disorder for differentially expressed venom proteome identified genes. AIUPred was used to predict the disorder along the protein sequences.

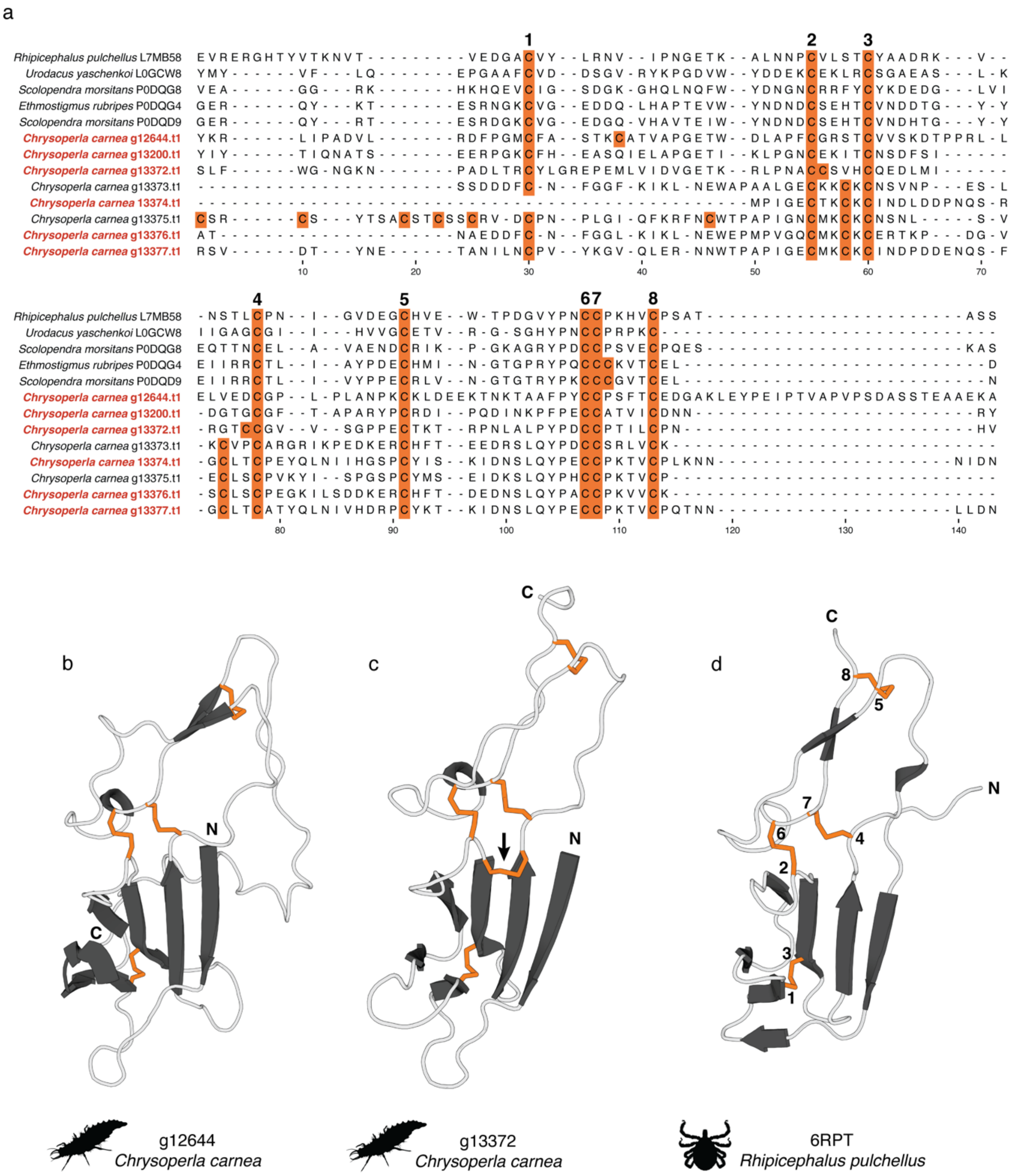

Figure S6. Venom proteome identified von Willebrand factor type C-like (SVWC) peptides, a common component of arthropod venoms. a) Alignment of different SVWC peptides found in other arthropod venoms (UniProt accession given in alignment) with *C. carnea* venom-identified SVWCs and those found in the annotation. **b**) Structure of a typical SVWC with conserved cysteine confirmation and **c**) a SVWC variant with an extra disulfide bond found in C. carnea. Structure predictions from ColabFold. **d**) Structure of a zebra tick SVWC (PDB: 6RPT).

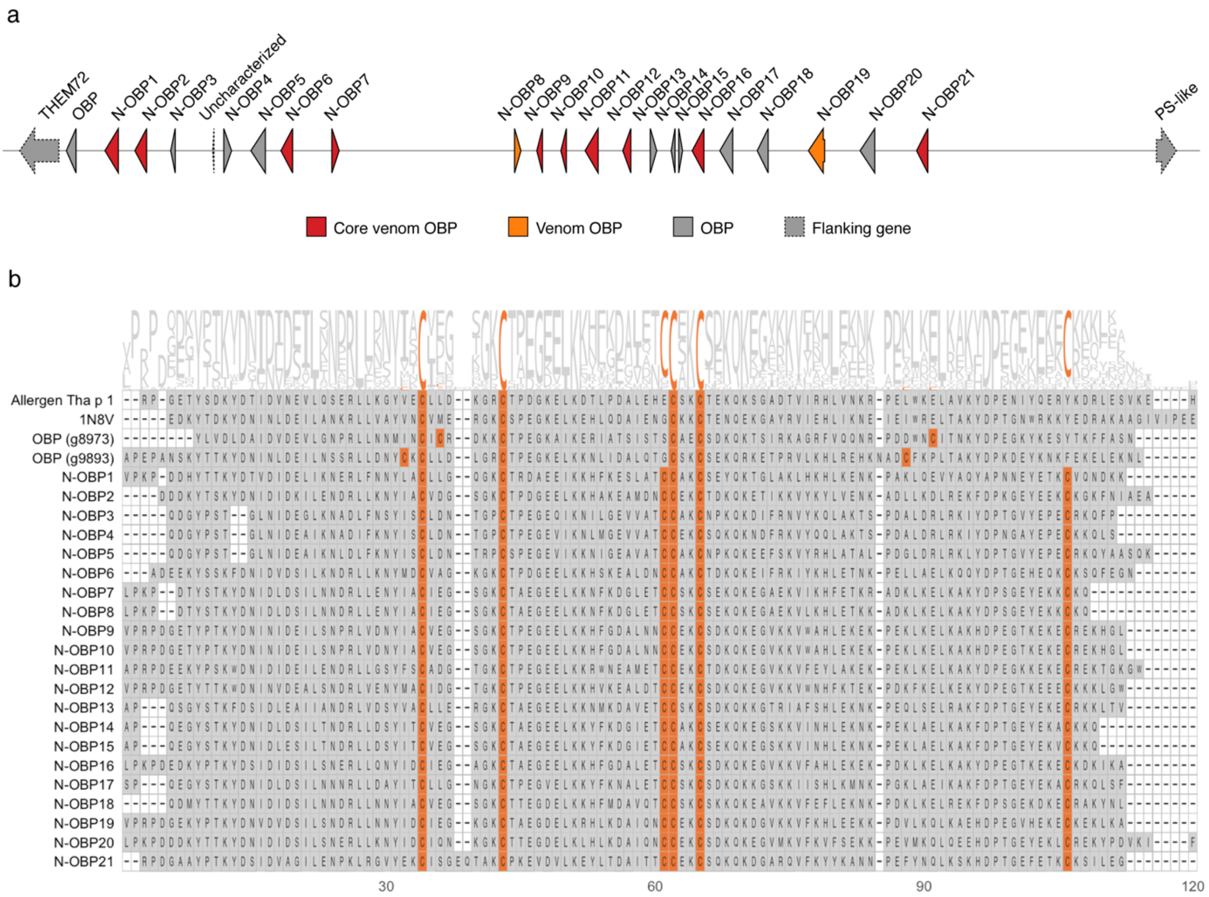

Figure S7. Genomic organization of an odorant binding protein (OBP) cluster identified in the venom of the *C. carnea*. a) The identified OBPs coloured according to if it was a core venom component (red), identified in the venom (orange) or not identified in the venom (grey). The flanking genes of this array are also highlighted. **b**) Sequence alignment of the identified OBPs including the representative structure for "Insect odorant-binding protein A10/Ejaculatory bulb-specific protein 3" (PDB: 1N8V) and the allergen Tha p 1 (UniProt: E0X9F6) from the pine processionary caterpillar *Thaumetopoea pityocampa*.

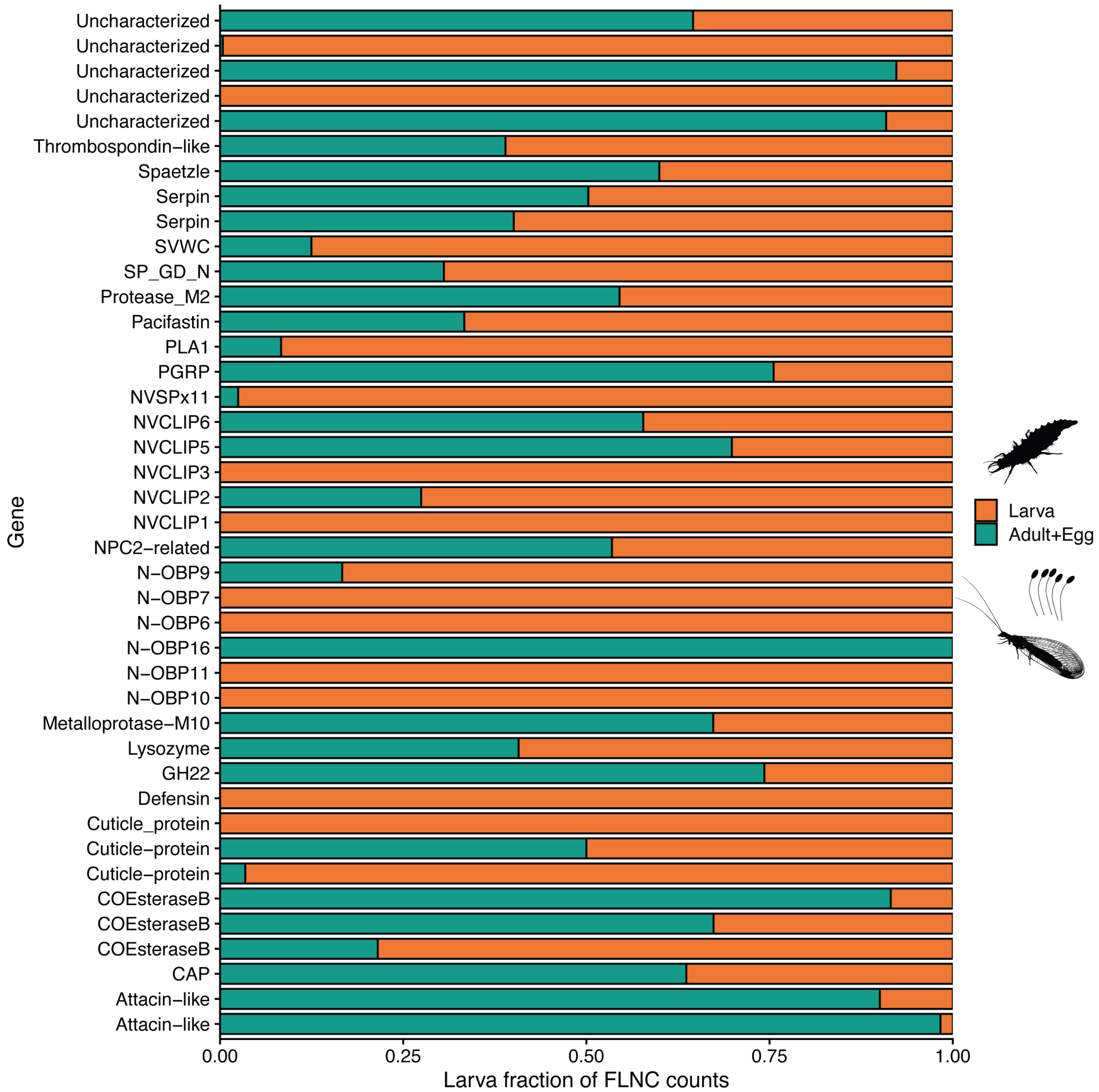

Figure S8. Larval fraction of FLNC reads from PacBio Iso-Seq data belonging to core venom component genes.

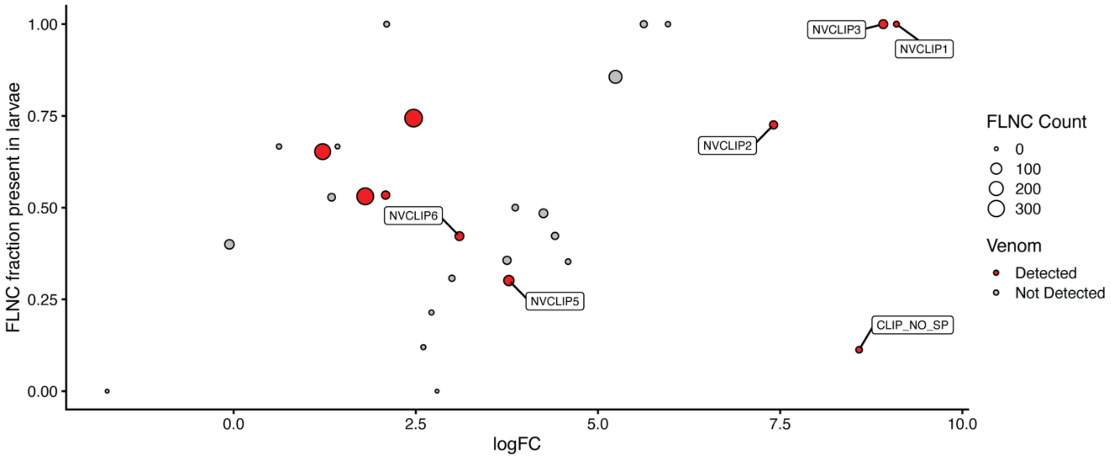

Figure S9. Gene expression of CLIP proteases and fraction of FLNC counts for those genes.

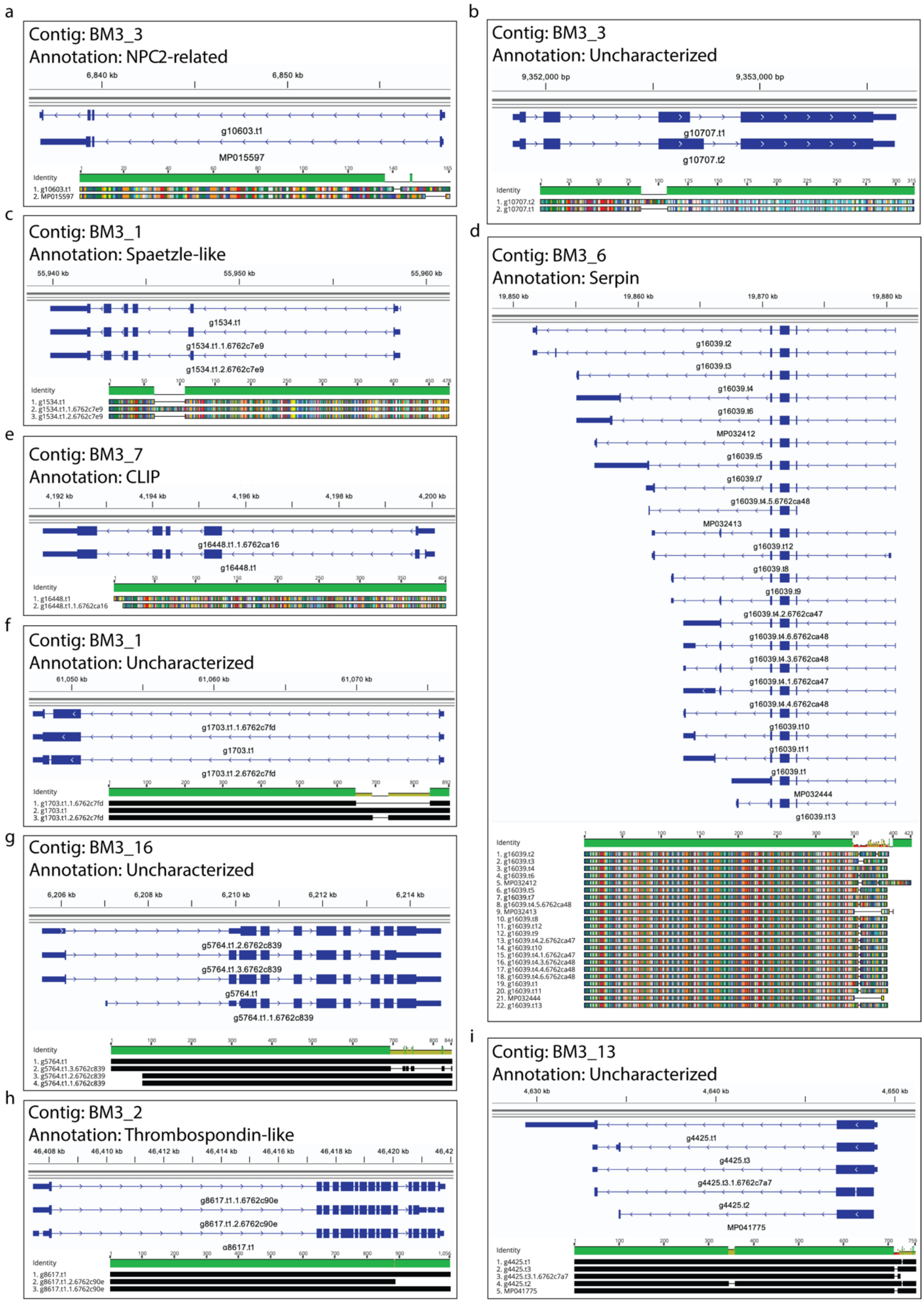

Figure S10. Splice variant visualization and alignment for the 9 core venom genes.

Supplementary tables

Table S1. BUSCO assessment of the genome assembly and gene annotations based on the Endopterygota_odb10 database (n=2124). For the gene annotation without isoforms, the longest isoform was chosen as a representative for each gene. The annotations were run with protein mode. Shorthands: S = Complete and Single-copy, D = Complete and Duplicated, F = Fragmented, M = Missing, n = Total BUSCO groups searched.

Data	Complete (%)	S (%)	D (%)	F (%)	M (%)
Genome assembly	97.3	95.8	1.5	1.1	1.6
Gene annotation	98.0	96.2	1.8	0.4	1.6
Gene annotation with isoforms	98.1	60.5	37.6	0.3	1.6

Table S2. Assembly comparison of the sequenced male green lacewing genome (BM3) to that of the available one inChrCarn1.1.

Measure	BM3	inChrCarn1.1 (GCA_905475395.1)
BUSCO (endopterygota_odb10)	C:97.3%[S:95.8%,D:1.5%],F:1.1%, M:1.6%,n:2124	C:95.9%[S:95.0%,D:0.9%],F:1.0%, M:3.1%,n:2124
Span (Mb)	596	560
Number of contigs	1312	399
Longest contig (Mb)	103	-
Contig N50 length (Mb)	37.1	67.8
Number of scaffolds	-	377
Scaffold N50 length (Mb)	-	94.4
Longest scaffold (Mb)	-	140

Table S3. BLASTp search results against the NCBI non-redundant protein database (nr; Title: All non-redundant GenBank CDS translations + PDB + SwissProt + PIR + PRF excluding environmental samples from WGS projects; Molecule type: Protein; Update date: 2025/05/18; Number of sequences: 920,902,381) for hemolysins reported in Fischer et al. (2024).

Query	Subject	Species	% Identity	E-value	Bit Score
CCARN_LC_C23769	KAL1139110.1	*Ranatra chinensis*	86.742	4.35e-160	456
CCARN_LC_C23769	ATU82495.1	*Lethocerus distinctifemur*	39.785	9.07e-41	151
CCARN_LC_C23769	QHB21510.1	*Platymeris rhadamanthus*	30.994	8.79e-18	94.0
CCARN_LC_C23769	ATU82700.1	*Pristhesancus plagipennis*	29.282	1.50e-07	63.9
CCARN_LC_C23769	ATU82699.1	*Pristhesancus plagipennis*	28.729	2.27e-06	60.5
CCARN_LC_C28028	KAL1139109.1	*Ranatra chinensis*	91.085	1.08e-142	411
CCARN_LC_C28028	ATU82504.1	*Lethocerus distinctifemur*	35.955	3.72e-20	98.6
CCARN_LC_C28028	ATU82699.1	*Pristhesancus plagipennis*	32.886	4.86e-06	59.3
CCARN_LC_C28028	ATU82700.1	*Pristhesancus plagipennis*	32.886	5.51e-06	59.3

Table S4. Matrix of orthogroup overlaps between the Neuroptera venom datasets. Each cell indicates the number of orthogroups shared between the corresponding pair of datasets.

Dataset	*C. carnea*	*C. carnea* (This study)	*E. nostras*
C. carnea	62	62	31
C. carnea (This study)	62	120	67
E. nostras	31	67	80

Table S5. Gene and orthogroup assignment summary for Neuroptera venom-identified genes using OrthoFinder. The table shows the number of genes per dataset, the number of genes assigned to orthogroups, unassigned genes, and unique orthogroups per dataset.

**Dataset**	**Number of Genes**	**Genes in Orthogroups**	**Unassigned Genes**	**Unique Orthogroups**
C. carnea (This study)	277	219	58	22
C. carnea	97	87	10	0
E. nostras	193	165	28	13
